# Trends of Water, Sanitation, and Hygiene (WASH) Research in Indonesia: A Systematic Review

**DOI:** 10.3390/ijerph19031617

**Published:** 2022-01-30

**Authors:** S. Satriani, Izana Saffana Ilma, D. Daniel

**Affiliations:** 1 Department of Environmental Science, The Graduate School, Universitas Gadjah Mada, Yogyakarta 55281, Indonesia; satriani.pmu@mail.ugm.ac.id (S.S.); izana.saffana.i@mail.ugm.ac.id (I.S.I.); 2 Department of Water Management, Faculty of Civil Engineering and Geosciences, Delft University of Technology, 2628 CN Delft, The Netherlands; 3 Department of Health Behaviour, Environment, and Social Medicine, Faculty of Medicine, Public Health and Nursing, Universitas Gadjah Mada, Yogyakarta 55281, Indonesia

**Keywords:** water, sanitation, hygiene, WASH, systematic review, Indonesia

## Abstract

This study provides an overview of water, sanitation, and hygiene (WASH) research trends in Indonesia from 1975 until April 2021. The systematic review compiled 272 articles related to the Sustainable Development Goals 6.1 and 6.2 in Indonesia, which were published in the Web of Science and Scopus databases. The results showed that the water-related topic (41%) was discussed more often than sanitation (22%) or hygiene (13%). Furthermore, the social theme (39%) was dominantly found in all these articles, mostly finding determinants of WASH-related behavior. However, few WASH implementation studies or behavioral change interventions were recorded in Indonesia, suggesting a gap between science and policy or implementation. On the other hand, hygiene-related topics (14%) and WASH-related financial themes (6%) were the least studied in Indonesia. Combinations of topics (23%) and themes (15%) were also often conducted in Indonesia, suggesting that WASH researchers started to recognize the need to analyze WASH problems holistically, i.e., from multiple perspectives. In addition, the distribution of WASH research was still dominated in the central part of Indonesia, whereas the WASH-related problems, i.e., poor WASH services, and behavior, often occur in this area. This study also offers some research gaps, both in terms of topics, themes, and regional distribution, that need to be considered for the design of future WASH research in Indonesia.

## 1. Introduction

Access to water, sanitation, and hygiene remains a global public health concern, as stated in the Sustainable Development Goals 6.1 and 6.2. Despite substantial increases in access to water, sanitation, and hygiene (WASH) services over the past thirty years, an estimated two billion people worldwide still lack access to safely managed drinking water, 3.6 billion people lack safe sanitation, and 2.3 billion people around the world lack basic hygiene services [1]. Poor WASH services can weaken health systems, threatens health security, and weigh on the economy. Therefore, appropriate WASH services improve the quality of life and fulfill human rights. WASH’s contribution is not only in the health sector, but also has implications for livelihoods, school attendance, and dignity and helps create resilient communities living in healthy environments [2]. This applies to developing countries, one of which is Indonesia, which is the fourth most populated country in the world and will get a demographic bonus in 2045 [3].

In 2011, the access for basic WASH services in Indonesia was around 55% and 56% for drinking water and sanitation services, respectively [4]. In 2020, the National Planning and Development Agency (“Bappenas” in Bahasa) adjusted standards for improving the quality of drinking water, settlements, and national sanitation according to the Sustainable Development Goals (SDGs) standards from “decent” to “safe” access [5]. With these efforts, in 2020, access to basic water, sanitation, and hygiene services increased to 92%, 86%, and 94%, respectively [1].

The WASH-related study is essential for supporting the acceleration of achieving SDG 6.1 and 6.2 in Indonesia. The study’s results can be used to critically review and monitor the current progress, develop evidence-based policy, or find causes of WASH-related phenomena [6]. Furthermore, WASH studies in Indonesia should cover all topics in WASH, including water, sanitation, and hygiene, or combinations of them, so there is adequate scientific support in achieving SDG 6.1 and 6.2 in Indonesia. The distribution of topics could also indicate the past and current research interests of WASH researchers in Indonesia. For example, despite its importance, few studies conducted in hygiene may indicate that this topic is underestimated or attracts little attention from WASH researchers in Indonesia.

Moreover, since there is a variation of WASH services, access, and also problems in different parts of Indonesia [7], it is important that WASH studies can be conducted locally to give an overview of the local situation, e.g., challenges or problems in a district or provincial levels, and then provide recommendations to solve those problems.

Therefore, it is essential to understand the past and current trends of WASH research in Indonesia. This can guide future WASH research in Indonesia. For example, by knowing the inequalities of the geographical location of WASH research in Indonesia, one can plan to conduct WASH research in a location with few or no studies or information about the WASH condition. WASH researchers can also indicate knowledge gaps in specific themes that should be explored, e.g., whether we need more research on financial or social themes related to hygiene topics. To the best of our knowledge, no study systematically reviews the past and current trends of WASH research in Indonesia. This study aims to fill that gap. The systematic review method was conducted to assess WASH research trends in Indonesia.

## 2. Materials and Methods

The systematic review was carried out in accordance with the Preferred Reporting Items for Systematic Reviews and Meta-analysis (PRISMA) guidelines [8]. The literature review search strategy was to find all studies related to WASH in Indonesia published in Web of Science and Scopus until April 2021. The search keywords used were “water” OR “sanitation” OR “hygiene” OR “WASH” AND “Indonesia”.

The selected papers from each database were then inserted into Mendeley to exclude duplicate publications. Furthermore, the title of articles was checked manually and we excluded irrelevant topics. Articles included in the screening title are related to WASH keywords, e.g., drinking water, sanitary inspection, hand washing, latrine, water access, water quality, water supply, open defecation, water treatment, and fecal. We assumed that all articles are related to WASH are covered by those keywords. Hereupon, the abstract screening was carried out to identify articles that were included in SDGs 6.1 and 6.2. Afterward, articles that have met the inclusion criteria were included in the full-text review stage. The inclusion criteria included: (1) research main topic is related to WASH, especially SDGs 6.1 (water) and 6.2 (sanitation and hygiene); (2) access for the full paper to perform eligibility assessment; (3) full-text paper in English; and (4) research was conducted in Indonesia.

The following information was extracted from the included studies and recorded in Microsoft Excel: (1) WASH research topics, either water, sanitation, hygiene, or a combination. Combination here means that the article discusses more than one WASH topic, e.g., water and sanitation topics in one article; (2) Research theme categorized as Financial, Institution, environment, technical, social, or a combination. An article that covers more than one research theme was categorized into a combination theme; (3) Year of publication; (4) Keywords; and (5) Study location, i.e., province.

Descriptive analysis was carried out to specify information based on topic, themes, a trend of WASH research, and research region distribution. SPSS Statistic ver. 23 was used to analyze the association between research topics and research themes. Word cloud was also created to identify keywords that often appear in the title and abstract of the reviewed articles. The ArcGIS ver. 10.8 software was used to create the distribution of study locations in Indonesia.

## 3. Results and Discussion

### 3.1. Search Results

**Figure 1:**
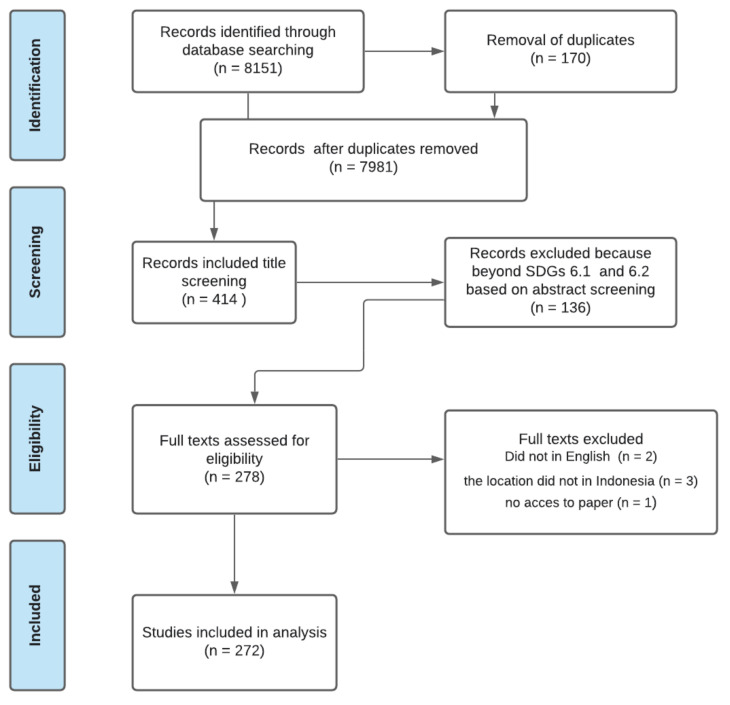
Diagram of the screening process and selected articles.

The systematic literature review retrieved 8151 articles from Scopus and Web of Science that were published before April 2021. Some duplicates were removed and resulted in 7981 articles. The title screening resulted in 414 studies related to WASH. In the next stage of abstract screening, 136 articles were excluded since those studies were not closely related to SDGs 6.1 and 6.2. At the full-text review stage, 278 articles were included. Of these, six articles were also excluded because those studies were not written in English (*n* = 2), were not located in Indonesia (*n* = 3), or the paper could not be accessed (*n* = 1). Thus, 272 studies were included in the final review process (Figure 1). More information on those studies can be found in the Supplementary Materials, e.g., explanation of themes (FIETS), type of study, and study scale.

### 3.2. Characteristic WASH Research by Topics, Themes, and Year

**Figure 2:**
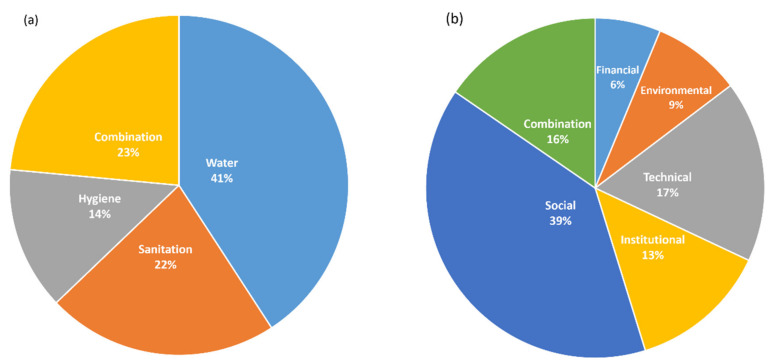
(**a**) Proportion of research topics and (**b**) Proportion of themes.

**Figure 3:**
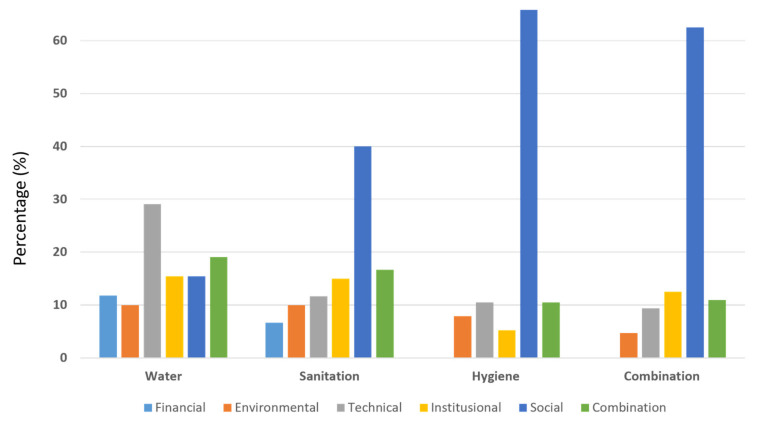
Percentage of themes in WASH topics.

The most discussed WASH topic in Indonesia was water (41%), followed by a combination topic (23%), and sanitation (22%) (Figure 2). The results also showed that 39% of the studies discussed the social theme. It dominates themes in hygiene (66%), combination (63%), and sanitation (40%) (Figure 3). The most common social research topic was the social-economic or behavioral determinants of the WASH access or behavior, e.g., [9,10]. This indicates that understanding the behavioral drivers of WASH practice is the core of WASH research in Indonesia.

**Figure 4:**
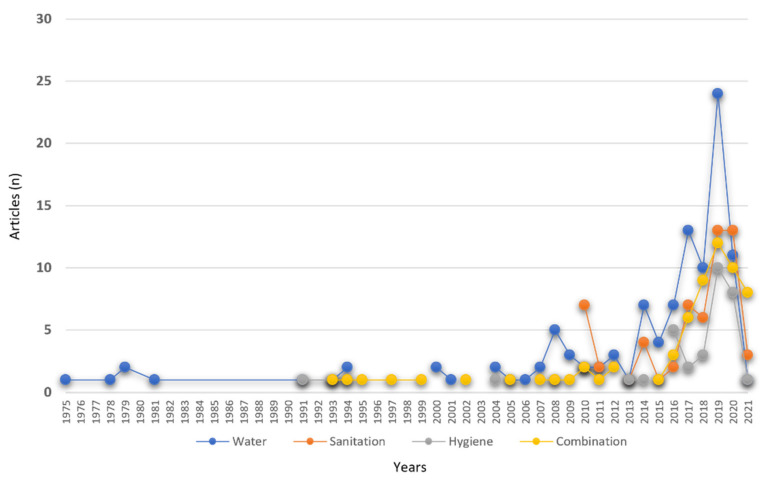
Growth of WASH research amount in Indonesia from 1975 to 2021.

Only 14% of the articles in the study discussed hygiene. The results show that hygiene has not been widely published in Indonesia, particularly during 1991–2004 (Figure 4). On the other hand, the outbreak of COVID-19 has provided a new paradigm where personal hygiene is the key element in controlling the spread of the virus [11]. Therefore, this finding suggests the need to conduct more hygiene-related research to provide a better understanding of hygiene conditions in Indonesia. 

Another theme with the highest percentage was the technical theme (18%) (Figure 2). However, this only applied to the water-related topic articles (29%) (Figure 3). These articles mainly discussed water treatment, e.g., [12]. The percentage of institutional themes was 13–15% in water, sanitation, and combination articles, but only 5% in hygiene-related topic articles. Only two articles of the institutional themes were in the hygiene topic, which discussed low resource settings for clinicians contacted with patients without hand hygiene in rural hospitals [13] and the evaluation of the children under two years old program (“Baduta Program” in Bahasa) [14].

The financial theme had the smallest proportion in WASH research in Indonesia (6%). Several studies link poverty with access to WASH services, e.g., [15]. Most of the financial-related themes in the water topic were about water service tariff and willingness to pay (12% of all financial-related themes), e.g., [16]. Financial-related themes in sanitation (7%) were about selecting affordable sanitation systems for the community, e.g., [17] (Figure 3). On the other hand, it was found that none of the hygiene articles addressed the financial theme. In a report published by GLAAS in 2019, Indonesia already has policies and plans to develop cost estimates for WASH plans covering aspects of drinking water and sanitation, but not hygiene aspects [18], whereas, there is a cost needed for hygiene facility, e.g., to buy soap or install handwashing facility in a public place. This suggests a knowledge gap in financial research on the topic of hygiene in Indonesia.

The first recorded publication of WASH research in Indonesia was in 1975, but then no article was published between 1981–1991. WASH research in Indonesia has been published regularly since 1991 until now, except in 1992, 1998, and 2003. Significantly, WASH research in Indonesia continues to grow in the 2015–2020 period. Five articles before 1990 were published on water topics. Moreover, the number of articles related to the water topics reached its peak in 2019, i.e., 24 articles. Water-related topics were dominant between 1975–1981, but then combination-related topics were dominant between 1990–2000 (Figure 4).

In contrast to water topics, there were year gaps in sanitation and hygiene topics. The study on sanitation had not been published until 2010 and became the most discussed topic in 2010 compared to other topics. We suspect this is related to the determination of sanitation as one of the main targets in Indonesia’s 2010–2014 Medium Term Development Plan (“RPJMN” in Bahasa). The national policy has, previously, indirectly driven the sanitation research in Indonesia. Furthermore, despite there being hygiene research in 1991, there was a long wait until the second study on the hygiene topic in 2004. Hygiene research started to increase in 2016, which may have been driven by the SDGs implementation in Indonesia.

**Figure 5:**
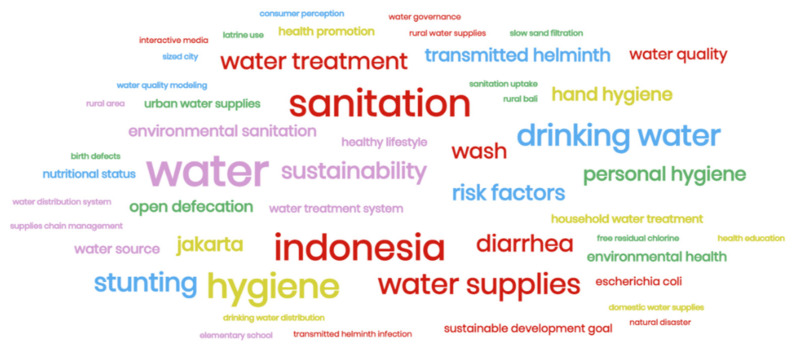
The word cloud of WASH research in Indonesia.

The title and abstracts of all articles were analyzed to identify the most frequent keywords in Indonesia’s WASH research. Drinking water, water supplies, stunting, diarrhea, and sustainability were the top 5 keywords (Figure 5). Since 40% of the studies were on water topics (Figure 1), terms related to water dominate the keyword identification results. Drinking water has become a widely discussed sub-topic, e.g., safe drinking water sources, treatments to ensure good water quality, and selection for piped and bottled water. The word cloud brings out some terms related to diseases, e.g., stunting and transmitted helminth. This indicates that WASH research in Indonesia began to be linked with the health issue.

### 3.3. Geographical Distribution of WASH Research in Indonesia

**Figure 6:**
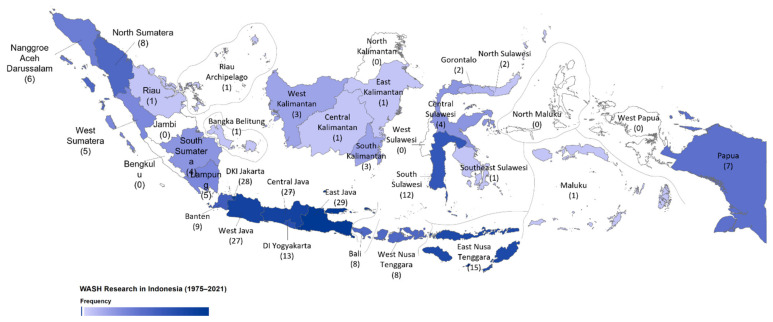
Provincial distribution of WASH research in Indonesia from 1975 to April 2021.

Identification of WASH research locations in Indonesia was categorized into four groups. The first group was multi-country research in which Indonesia was included as one of the study countries. This group primarily discusses sanitation and social topics as dominant themes, including financial and social, e.g., [17]. The second group was studies located in Indonesia, but which do not mention the exact province or location. The second group comprises 56 articles, which mainly discussed combination topics and social themes, e.g., [19]. The third group was studies conducted in several provinces. These studies were often related to sanitation topics and social themes with 10 articles, e.g., [20]. The last group was studies conducted only in one specific province, with 203 articles, e.g., [21] (Figure 6), or equal to 74.5% of the total reviewed articles.

Moreover, if we look at the research location at the provincial level in Indonesia, the results showed that there is inequality in the WASH research conducted in Indonesia. Most of the research was conducted in the provinces located in Java Islands (Figure 6). The province with the most frequent WASH research location in Indonesia was West Java with 29 articles, e.g., [22], Jakarta with 28 articles, e.g., [16], Central Java with 27 articles, e.g., [23], and East Java with 27 articles, e.g., [24]. All those provinces were located on the Java Island which has the best research facilities in Indonesia. Studies conducted in East Java mainly discussed sanitation topics, while water topics dominated studies conducted in Jakarta, Central Java, and West Java. For the theme of the articles, East Java and West Java were dominated by social themes, Jakarta with technical and social themes, and Central Java with combination themes.

On the other hand, in Central Indonesia, East Nusa Tenggara had the highest WASH research with 15 articles, and in Eastern Indonesia, Papua province had the highest WASH research, i.e., 7 articles. Provinces with a small number of WASH research in Indonesia were South Kalimantan, with three articles, e.g., [25], and West Kalimantan, i.e., 3 articles, e.g., [26], and Gorontalo, i.e., 2 articles, e.g., [27]. Provinces with only one article were Bangka Belitung [28], East Kalimantan [29], Riau [30], Riau Archipelago [31], Central Kalimantan [25], Southeast Sulawesi [32], North Sulawesi [33], and Maluku [34]. Lastly, there was no WASH research recorded in six provinces between 1975–April 2021, i.e., Jambi, Bengkulu, North Maluku, and West Papua.

### 3.4. Recommendation for Future WASH Research in Indonesia

This systematic review provides some recommendations for future WASH research, in terms of topic, theme, and area. The hygiene-related research topic should be conducted more often in Indonesia. There are various contexts of hygiene that are potentially explored in Indonesia, e.g., hygiene in school, emergencies settings, healthcare facilities, menstrual hygiene, etc. These contexts can complement the current context in hygiene research in Indonesia, which focused mostly on personal and household hygiene. Furthermore, the COVID-19 pandemic has made hygiene an important discussion around the world and a daily necessity to prevent the spread of the COVID virus. We believe that this is a nice momentum to boost hygiene-related research in Indonesia. Another knowledge gap is that there is no hygiene research related to the financial theme. Future hygiene studies in Indonesia should address this topic, especially to escalate hygiene services, e.g., handwashing facilities, in vulnerable areas, e.g., emergency, school, and rural areas. 

The social theme is common in the WASH research in Indonesia, which is dominated by finding the drivers or determinants of WASH practice. However, based on our review, there were only two scientific articles, e.g., [10,35] that discuss behavior change interventions in Indonesia, whereas it is important to learn the lessons on how to change community behavior in WASH. Furthermore, based on our observation, behavioral change intervention is one of the cores of activity of many WASH-related non-governmental organizations (NGOs) in Indonesia. However, it could be that the NGOs do not collaborate closely with academia in conducting their WASH project, which results in very few scientific articles on the behavioral change intervention in WASH coming from Indonesia. Therefore, we suggest a collaboration between WASH NGOs and academia in conducting implementation research in Indonesia, e.g., a baseline study, follow-up by the behavioral change intervention, and the project outcomes or evaluation are reported in a scientific journal. This is also to ensure that any WASH research does not merely end up in a scientific article but can be used to design relevant policy or intervention.

Financial theme research was rarely conducted in Indonesia. Financial-related studies are needed to develop strategies to enhance the practice of and access to WASH services, especially among the poor, e.g., assessing the willingness to pay or find cost-efficient technology that can be implemented in society at a low cost. For example, a recent study conducted in less developed and rural Indonesia shows that many households in such areas could not afford WASH technologies, e.g., the latrine [36], and a financial-related study is needed to find the solution for this.

An adequate number of the combination-related topics (23.4%), e.g., water and sanitation, and themes (15%), e.g., social and financial. Based on our experience, the result suggests several things. First, WASH researchers in Indonesia start to realize that all topics in WASH are correlated, e.g., access to water is correlated with the sanitation practice. Moreover, considering the F-diagram that exposure to a pathogen can be through multiple pathways [37], the analysis of the provision of safely managed water services should be accompanied. Second, the combination-related themes suggest that WASH researchers in Indonesia try to see the WASH problem from multiple perspectives, i.e., multi-disciplinary. Research themes are categorized as financial, institution, environment, technical, social, or combination (FIETS). Daniel et al [36] show how financial, institution, environmental, technology, and social (FIETS) are interrelated in the WASH sector. Neglecting one aspect will hinder us to understand fully the problem. Moreover, WASH experts and practitioners have recognized that the WASH problem is complex [38,39]. Future WASH studies in Indonesia should then address the complexity of WASH conditions in a specific area by analyzing multiple WASH topics, e.g., water, sanitation, and hygiene, and also multiple themes, e.g., FIETS.

While conducting multiple WASH topics and themes can give a holistic view of the WASH situation in a specific area, it has some potential challenges. First, the WASH researchers in Indonesia, which often come from the field of environmental science or engineering or public health, need to involve people from different disciplines, e.g., economic or psychology. Reducing this “discipline barrier” is still a challenge in conducting multidisciplinary research in Indonesia [40]. Second, the construction of knowledge in Indonesian education is largely formed within the boundaries of discipline, which is a challenge in multidisciplinary research [41]. Even though the understanding of concepts and objectives has been equalized, different scientific perspectives have resulted in differences in answering research problems, for example when conducting screening papers.

There is inequality in WASH research in Indonesia, in which areas outside Java tend to be under-researched. There were four provinces without any WASH research recorded, i.e., Jambi, Bengkulu, North Maluku, and West Papua. This could be due to the limitations of technical and human resources needed to conduct WASH research and also geographical conditions that are difficult to reach. The government could provide research grant opportunities to conduct WASH studies in these areas to tackle the technical resources problem. Moreover, universities located in Java Island, i.e., which often have better resources, can also consider these areas as their future study location to overcome the human resources issue. They can collaborate and help local universities to conduct WASH research. All these actions are needed to increase the number of WASH research outside Java, i.e., solve WASH problems and also monitor the progress of SDG 6.1 and 6.2 in all provinces in Indonesia.

Factors that influence the WASH conditions, behaviors, or services vary depending on contexts or settings and we could not simply extrapolate the research results, e.g., apply the same recommendations, in one context or setting to others [42]. Thus, the WASH intervention must be adapted to local circumstances, highlighting the need for local WASH research. Our systematic review found that three-fourths of the reviewed articles were located in one province only, suggesting that those studies try to find solutions for a local WASH problem. This also implies that there are many potential WASH studies that can be conducted in Indonesia, considering various WASH topics, themes, and locations. 

The review also shows that there are knowledge gaps of WASH research among vulnerable communities, e.g., disabilities, indigenous, and remote communities. Thus group has the right to full and effective participation in all aspects of life. Its manifestation can be related to the fulfillment of accessibility both in the physical environment, transportation, information and communication, and access to other facilities and services that are open or provided to the public, both in urban and rural areas [43]. They are often neglected and have poor access to WASH services [44]. Future WASH research should target these communities to understand their problems and ensure that there are no communities or areas left behind in achieving SDG 6.1 and 6.2 in Indonesia. 

Other potential WASH research in Indonesia is related to WASH services in school, emergency settings, and healthcare facilities. Universal access to WASH in SDGs 6.1 and 6.2 covers all settings, including households, schools, health facilities, workplaces, and public places [45]. A report indicates that the basic WASH services at schools in Indonesia in 2019 were 72.73% for water, 40.40% for sanitation, and 58.86% for hygiene. For health facilities in Indonesia, data shows that 80.17% have been covered by basic level WASH services [46]. Our review shows that there is only one WASH study in the school setting in Indonesia [47], and one study related to healthcare facilities, i.e., discussed the hygiene of health workers [13]. Additionally, there is only one study related to the emergency setting in Indonesia [27]. We think that more research is needed to understand how to enhance access to WASH services in these settings.

Menstrual hygiene is another knowledge gap that starts to gain interest among WASH researchers in Indonesia [48]. Menstrual hygiene management (MHM) is still limited in Indonesia, besides that in most primary schools, hygiene in sanitation facilities is still lacking [49]. Girls find it challenging to access WASH facilities that can be used and are suitable for menstruation, e.g., in school, whereas, one of the problems that often occur in school-age girls who have reached the age of puberty do not attend school during menstruation [50]. All these research areas attract the attention of the global WASH practitioners in the past years and are potentially conducted in future WASH research in Indonesia.

There are some limitations of this review. First, due to the wide scope of this study, the knowledge gap in this WASH research is also not very detailed, i.e., there are no specific and detailed knowledge gaps in each topic, theme, and region. A future review study may focus on a smaller scope so one can assess the knowledge gaps in more detail, e.g., the review can focus only on the water topic and discover knowledge gaps on this topic. Second, we do not assess the risk of bias or quality of the reviewed studies. Thus, the topic or theme may have been studied before but we do not the quality of the study. Therefore, if one wants to conduct a study on the same topic or theme, we suggest studying carefully the previous study and designing their study on top of the previous study, by considering also the risk of bias and quality of the previous study. More information about all reviewed studies in this article can be found in the Supplementary Materials. Finally, this study can be seen as a starting point by scholars, especially in Indonesia, to design more comprehensive WASH research in Indonesia.

## 4. Conclusions

This review study discusses 272 articles on WASH-related research in Indonesia published before April 2021. The result shows that the most frequent research topic was water, while the dominant research theme in Indonesia is the social theme. The technical theme, e.g., water supply and water treatment, was dominant in the water topic, while the social theme was dominant in the sanitation, hygiene, and combination topics. Almost half of the total studies were conducted in Java Island. There are few records of implementation research or behavioral change intervention in the WASH studies in Indonesia. This implies that past WASH research in Indonesia often stopped only at finding behavioral determinants. Future WASH research in Indonesia can consider hygiene and WASH-related to financial research topics, researching the indigenous and remote populations or areas, in school, emergency, and health-care facility settings, and also related to menstrual hygiene. Research on these topics can enrich our understanding of the current WASH situations in Indonesia.

## Data Availability

The data is contained within the article or Supplementary Material.

## Supplementary Materials

The following supporting information can be downloaded at: https://www.mdpi.com/article/10.3390/ijerph19031617/s1, Table S1: Definition each theme in research; Table S2: Article used in The Analysis of WASH Research Trends in Indonesia; Table S3: PRISMA checklist.
