# Supplementary material for: Trends of Water, Sanitation, and Hygiene (WASH) Research in Indonesia: A Systematic Review
Source: Int J Environ Res Public Health. 2022 Jan 30;19(3):1617. doi: 10.3390/ijerph19031617 (PMC8835571; doi:10.3390/ijerph19031617)
Supplement: Supplementary file 1 [file ijerph-19-01617-s001.zip › ijerph-1493956-supplementary.pdf]

Supplementary material for :

Trends of water, sanitation, and hygiene (WASH) research in Indonesia: A systematic review

S. Satriani[a], Izana Saffana Ilma[a], D. Daniel[b*]

[1] Department of Environmental Science, The Graduate School, Universitas Gadjah Mada, 55281 Yogyakarta, Indonesia; satriani.pmu@mail.ugm.ac.id; izana.saffana.i@mail.ugm.ac.id

[2] Department of Water Management, Faculty of Civil Engineering and Geosciences, Delft University of Technology, 2628 CN Delft, The Netherlands

[3] Department of Health Behaviour, Environment, and Social Medicine, Faculty of Medicine, Public Health and Nursing, Universitas Gadjah Mada, 55281 Yogyakarta, Indonesia

* Correspondence: d.daniel@tudelft.nl; daniel01@ugm.ac.id

*Corresponding author: daniel01@ugm.ac.id

The total article used in this systematic review is 272 articles (Tabel 1) derived from the Scopus and Web of Science databases. This study provides an overview of water, sanitation, and hygiene (WASH) research trends in Indonesia from 1975 until April 2021 and show an increasing number of WASH articles published each year. Research topics on water and research themes on social are the most dominant topics and themes compared to others. The distribution of WASH research locations in Indonesia is unequal and only focused on western Indonesia. This finding is expected to be taken into consideration for designing WASH research in the future.

Table S1. Definition each theme in research.

Theme	Definition
Financial	The financial theme discusses the provision of products and services related to guaranteed water, sanitation, and hygiene that are financed locally (e.g., taxes, regional levies, local financing) and do not depend on external (foreign) subsidies.
Environment	WASH in the environmental context includes an integrated and sustainable approach to water and waste (-water) management of streams and resources.
Institutional	This theme encompasses WASH systems, institutions, policies, and procedures.
Technical	The technical theme covers the use of technology or hardware in an attempt to access WASH.
Social	The social context in WASH discusses related to demand-driven, inclusive (equality), gender equality, culturally sensitive, and needs-based.
Combination	This theme is a combination of several themes.

Table S2. Article used in The Analysis of WASH Research Trends in Indonesia.

* Research Topic : 1 = Water ; 2 = Sanitation ; 3 = Hygiene; 4 = Combination,

** Research Theme : 1 = Financial ; 2 = Environmental ; 3 = Technical; 4 = Institutional ; 5 = Social ; 6 = Combination

*** "-" in keyword means that there is no keyword in the article.

NO	TITLE	AUTHOR	YEAR	KEYWORD	LOCATION	STUDY SCALE	TYPE OF THE STUDY	RESEARCH TOPIC	RESEARCH THEME
1	Water-Supply Problems and Developments in Indonesia	Notosugondo, H.	1975	-	Indonesia	National	Ideas, Editorials, Opinions	1	4
2	Water Supply Problems in Jakarta, Indonesia	Nazir, I.	1978	-	Jakarta	Provincial	Evaluation study	1	3
3	Faecal pollution of surface waters in Jakarta	Gracey, M., Ostergaard, P., Adnan, S.W., and Iveson, J.B.	1979	-	Jakarta	Provincial	Cohort Study	1	3
4	Polluted water and childhood diarrhoea in Jakarta, Indonesia	Gracey, M.	1979	-	Jakarta	Provincial	Cross-sectional study	1	2
5	Banyusidi village water supply: A case study of project implementation and utilization in rural Indonesia	Williams, G. and Sirait. D.	1981	-	Central Java	Provincial	Evaluation study	1	6
6	Evaluating 3 Simple Methods to Asses The MIicrobial Quality of Drinking-Water in Indonesia	Kromoredjo, P. and Fujioka, R. S.	1991	-	South Kalimantan	Provincial	Experimental design	1	3
7	Hand-Washing Reduces Diarrhea Episodes - A Study in Lombok, Indonesia	Wilson, J. M., Chandler, G. N., Muslihatun, and Jamiluddin.	1991	-	West Nusa Tenggara	Provincial	Controlled Clinical Trials	3	5
8	Health, Safe Water and Sanitation - A Cross-Sectional Health Production Function for Centrak Java, Indonesia	Wibowo, D., and Tisdell, C.	1993	-	Central Java	Provincial	Cross-sectional study	4	3
9	Rent-Extracting Behavior by Multiple Agents in The Provision of Municipal Water Supply - A Study of Jakarta, Indonesia	Lovei, L. and Whittington, D.	1993	-	Jakarta	Provincial	Evaluation Studies	1	4
10	Sustained Improvements in Hygiene Behaviour Amongst Village Women in	Wilson, J .M., and Chandler, G. N.	1993	-	West Nusa Tenggara	Provincial	Controlled Clinical Trials	3	3

	Lombok, Indonesia								
11	Water markets, market reform and the urban poor: Results from Jakarta, Indonesia	Crane, R.	1994	-	Jakarta	Provincial	Cross-sectional study	1	1
12	Personal and domestic hygiene and its relationship to the incidence of diarrhoea in south Sumatera.	Aulia, H., Surapaty, S.C., Bahar, E., Susanto, T.A., Roisuddin, Hamzah, M., and Ismail, R.	1994	Diarrhoeal Diseases, Hygiene Behaviour, Sociodemographic Characteristic, Drainage system, Defaecation, Sanitation	South Sumatera	Provincial	Controlled Clinical Trials	3	5
13	Water Preparation Practices in South Kalimantan, Indonesia	Prihartono, N., Adisasmita, A., Costello, C., Damayanti, R., Prasetyo, S., and Syarif, S.	1994	Diarrhoea, Water, Water supply, Sanitation	South Kalimantan	Provincial	Cross-sectional study	4	5
14	Cost-Effectiveness of Water Supply Technologies in Rural Indonesia - Evidence from Nusa Tenggara Barat	Perkins, F.	1994	-	West Nusa Tenggara	Provincial	Cost-Benefit Analysis	1	1
15	2 Years Investigation of Epidemic Hepatitis-e Virus Transmission in West Kalimantan (Borneo), Indonesia	Corwin, A., Jarot, K., Lubis, I., Nasution, K., Suparmawo, S., Sumardiati, A., Widodo, S., Nazir, S., Orndorff, G., Choi, Y Tan, R., Sie, A., Wignall, S., Graham, R., and Hyams, K.	1995	hepatitis E virus, epidemiology, serology, Indonesia	West Kalimantan	Provincial	Cross-sectional study	4	3

16	Enterotoxigenic Escherichia coli diarrhea among young children in Jakarta, Indonesia	Richie, E., Punjabi, N.H., Corwin, A., Lesmana, M., Rogayah, I., Lebron, C., Echeverria, P., and Simanjuntak, C.H.	1997	-	Jakarta	Provincial	Causality	4	5
17	The unique riverine ecology of hepatitis E virus transmission in South-East Asia	Corwin, A. L., Tien, N. T. K., Bounlu, K., Winarno, J., Putri, M. P., Laras, K., Larasati, R. P., Sukri, N., Endy, T., Sulaiman, H. A., Hyams, and Kenneth C	1999	Indonesia; Laos; South-East Asia; Viet Nam; ecology; epidemiology; hepatitis E virus; hepatitis E virus antibodies; prevalence	Indonesia, Vietnam	Multi countries	Cross-sectional study & Case control study	4	2
18	Block rate pricing of water in Indonesia: An analysis of welfare effects	Rietveld, P., Rouwendal, J., and Zwart, B.	2000	-	Central Java	Provincial	Cost-Benefit Analysis	1	1
19	Nanofiltration for drinking water production from deep well water	Khalik, A. and Praptowidodo, V. S.	2000	Deep well water; Nanofiltration	East Kalimantan	Provincial	The conceptual design system with a NF	1	3
20	Poor food hygiene and housing as risk factors for typhoid fever in Semarang, Indonesia	Gasem, M. H., Dolmans, W. M. V., Keuter, M., and Djokomoeljanto, R.	2001	typhoid fever, case-control, risk factors, Indonesia	Central Java	Provincial	Case-control study	1	5

21	First documented outbreak of hepatitis E virus transmission in Java, Indonesia	Sedyaningsih Mamahit, E. R., Larasati, R. P., Laras, K., Sidemen, A., Sukri, N., Sabaruddin, N., Didi, S., Saragih, J. M., Myint, K. S. A., Endy, T. P., Sulaiman, A., Campbell, J. R., and Corwin, A. L.	2002	Indonesia; hepatitis E virus; infections; outbreak; polymerase chain reaction; prevalence; risk factors; serology	East Java	Provincial	Cross-sectional study	4	3
22	Potable water source and the method of garbage disposal in lowering the risk of diarrhea	Suriyasa, P., Balgis, Saptono, R., and Hapsari, M.I.	2004	diarrhea, potable water, garbage disposal, family health potential index, poor family	Indonesia	National	Cross-sectional study	1	5
23	Mercury concentrations in the community drinking water sources around Manado City, North Sulawesi, Indonesia	Limbong, D., Kumampung, J., Rumengan, I. F.M., Arai, T., and Miyazaki, N.	2004	-	North Sulawesi	Provincial	Cross-sectional study	1	2
24	Risk factors for transmission of foodborne illness in restaurants and street vendors in Jakarta, Indonesia	Vollaard, A. M., Ali, S., Van Asten, H., Ismid, I. S., Widjaja, S., Visser, L. G., Surjadi, C., and Van Dissel, J. T.	2004	-	Jakarta	Provincial	Cross-sectional study	3	2
25	Households' valuation of domestic water	Yusuf, A. A. and	2005	-	Indonesia	National	Cost-Benefit	1	1

	in Indonesia: Revisiting the Supply Driven Approach	Koundouri, P.					Analysis		
26	Disaster relief and initial response to the earthquake and tsunami in Meulaboh, Indonesia	Lee, V. J., Low, E., Ng, Y. Y., and Teo, C.	2005	Healthcare delivery, Natural disasters, Public health, Relief work Introduction	Aceh	Provincial	Empirical Research	4	2
27	Monitoring of human enteric viruses and coliform bacteria in waters after urban flood in Jakarta, Indonesia	Phanuwan, C., Takizawa, S., Oguma, K., Katayama, H., Yunika, A., and Ohgaki, S.	2006	Coliform bacteria;E. coli; health risk; human enteric viruses; Jakarta; urban flooding	Jakarta	Provincial	Empirical Research	1	2
28	Factors associated with E. coli contamination of household drinking water among tsunami and earthquake survivors, Indonesia	Gupta, S.K., Suantio, A., Gray, A., Widyastuti, E., Jain, N., Rolos, R., Hoekstra, R.M., and Quick, R.	2007	-	Aceh and North Sumatera	Multi Provinces	Empirical Research	1	2
29	Trickle Down? Private sector participation and the pro-poor water supply debate in Jakarta, Indonesia	Bakker, K	2007	Development; Indonesia; Jakarta; Poverty; Privatization; Pro-poor; Water supply	Jakarta	Provincial	Ideas, Editorials, Opinions	1	4
30	The potential of nutrient reuse from a source-separated domestic wastewater system in Indonesia - Case study: ecological sanitation pilot plant in Surabaya	Malisie, A. F., Prihandrijanti, M., and Otterpohl, R.	2007	Anthropogenic fertiliser; brownwater; ecological sanitation; greywater; vermicomposti ng;yellowwate	East Java	Provincial	Empirical Research	4	3

				r					
31	Cryptosporidiosis in children less than three years old in Ciliwung Riverside, Kampung Melayu Village, Jakarta, Indonesia	Soetomenggolo, H. A., Firmansyah, A., Kurniawan, A., and Trihono, P. P.	2008	cryptosporidium, children, prevalence, risk factor, clinical manifestations	Jakarta	Provincial	Cross-sectional study	4	2
32	Governance Failure: Rethinking the Institutional Dimensions of Urban Water Supply to Poor Households	Bakker, K., Kooy, M., Shofiani, N.E., and Martijn, E. J.	2008	water, infrastructure, poverty, access, Asia, Jakarta	Jakarta	Provincial	Evaluation study	1	4
33	Multi-decisions rating model: Establishing rescue policies for Regional Drinking Water Companies (PDAMs) in Indonesia	Peniwati, K. and Brenner, W.	2008	AHP; Drinking water companies; Indonesia; Multi-decisions model; Rating; Water Enterprise Association	Indonesia	National	Empirical Research	1	1
34	Housing sanitation and acute respiratory tract infection among undergraduate students in Indonesia.	Yudhastuti, R.	2008	housing sanitation; air quality; acute respiratory infection; university studentsIndonesia	East Java	Provincial	Cross-sectional study	2	6
35	Water quality of Angke River: Microbiological point of view	Tjampakasari, C. R. and Wahid, M. H.	2008	Total coliform, fecal coliform, most	Jakarta	Provincial	Cross-sectional study	1	3

				probability number, E.coli					
36	Assessing The Regulatory Model for Water Supply in Jakarta	Iwanami, M. and Nickson, A.	2008	concession; customer perception; regulation; urban water supply	Indonesia	National	Compares the regulatory system	1	4
37	Public private partnership for efficient and sustainable water supply development in Indonesia	Hoogsteen, K. J., van der Kolff, G., and Ruijter, J. A.	2008	drinking water supply, Indonesia, public-private partnership Introduction	Indonesia	National	Evaluation Study	1	1
38	Typical urban water supply provision in developing countries: a case study of Semarang City, Indonesia	Hadipuro, W. and Indriyanti, N. Y.	2009	Environmental problems; Groundwater extraction; Urban water supply	Central Java	Provincial	Qualitative Studies	1	6
39	Purchase of drinking water is associated with increased child morbidity and mortality among urban slum-dwelling families in Indonesia	Semba, R. D., de Pee, S., Kraemer, K., Sun, K., Thorne-Lyman, A., Moench-Pfanner, R., Sari, M., Akhter, N., and Bloem, M. W.	2009	Diarrhea; Drinking water; Morbidity; Mortality; Poverty	Indonesia	National	Cross-sectional study	1	5
40	Environmental Stewardship and the Humanitarian Aid Water and Sanitation Sector: Lessons from the 2004 Tsunami Disaster Response	Randall, J. J., Rand, E. C., Navaratne, A., and Hagos, Y.	2009	Humanitarian aid; environmental management; co-location; small-scale technology; watershed	Indonesia	National	Qualitative Studies	4	5

				management					
41	Operational experience with a micro hydraulic mobile water treatment plant in Indonesia after the ‘‘Tsunami of 2004’’	Garsadi, R., Salim, H. T., Soekarno, I., Doppenberg, A. F. J., and Verberk, J. Q. J. C.	2009	Disaster relief; Drinking water supply; Micro hydraulic mobile water treatment; Surface water treatment	Indonesia	National	Cohort Study	1	3
42	Hand hygiene in rural Indonesian healthcare workers: Barriers beyond sinks, hand rubs and in-service training	Marjadi, B. and McLaws, M.-L.	2010	Hand hygiene; Indonesia Low resource setting	Indonesia	National	Cross-sectional survey	3	4
43	Indonesia's water supply regulatory framework: Between commercialisation and public service?	Hadipuro, W.	2010	Water supply, regulation, commercialisation, public service, Indonesia	Indonesia	National	Evaluation Study	1	4
44	SANIMAS Approach and ISSDP's City-wide Sanitation Strategy (CSS)	Wibowo, J. S., and Legowo, H. B.	2010	Sanimas, ISSDP, PPSP, City-wide Sanitation Strategy, high risk areas, sanitation zoning	Indonesia	National	ISSDP approach	2	6
45	Integrated Assessment of the Feasibility of Community Based Sanitation Options: A Case Study From East Java, Indonesia	Starkl, M., Bisschops, I., Norstrom, A., Purnomo, A., and Rumiati, A.,	2010	biogas plant, constructed wetland, decentralized systems, ecosan, septic	East Java	Provincial	Cost benefit analysis	2	1

				tank					
46	Risk criticality and allocation in privatised water supply projects in Indonesia	Wibowo, A. and Mohamed, S.	2010	Indonesia; Private sector; Water supply; Criticality; Risk allocation	Jakarta	Provincial	Cross-sectional study	1	4
47	Treatment and Re-cycling of Sludge from Urban Decentralized Sanitation Facilities	Schmidt, A.	2010	urban sludge management, sludge disposal, treatment and re-use	East Java & West Sumatera	Multi Provinces	Experimental Study	2	3
48	Evaluation of community participation in the implementation of community-based sanitation systems: a case study from Indonesia	Roma, E. and Jeffrey, P.	2010	community participation, technology assessment, users' receptivity	Central Java	Provincial	Cross-sectional study	2	4
49	Assessment of The Health Status of Primary School Children Participating in School Feeding Program in Nusa Tenggara Barat and Nusa Tenggara Timur, Indonesia	Susilowati, D. and Sundari, S.	2010	school feeding, primary school children, anemia, knowledge attitude and pracltce	West Nusa Tenggara & East Nusa Tenggara	Multi Provinces	Randomized Controlled Trial	2	5
50	Gender- and Poor-inclusive Community-managed Sanitation and Hygiene in Urban Indonesia	Mozar, R. and Sijbesma, C.	2010	Community-based sanitation aspects: decentralized services, community management, gender, poor-inclusiveness,	Indonesia	National	MPA (Methodology for Participatory Assessment)	4	5

				hygiene promotion					
51	Sustainable Sanitation as a Part of an IWRM in the Karst Area of Gunung Kidul: Community Acceptance and Opinion	Nayono, S., Singer, M., Lehn, H., and Kopfmueller, J.	2010	Sustainable sanitation; karst area; urine diverting toilet; composting toilet	Yogyakarta	Provincial	Case-control study	2	3
52	Food and Personal Hygiene Perceptions and Practices among Caregivers Whose Children Have Diarrhea: A Qualitative Study of Urban Mothers in Tangerang, Indonesia	Usfar, A. A., Iswarawanti, D. N., Davelyna, D., and Dillon, D.	2010	hygiene, perception, practice, diarrhea, mothers	Banten	Provincial	Qualitative study	3	5
53	Teachers' Beliefs and Perceptions of Integration and Elicitation of Human Values in Water Education in Some Southeast Asian Countries	Parahakaran, S.	2010	-	Teachers surveyed were from Indonesia, Thailand and Lao PDR	Multi countries	Qualitative study	4	5
54	Empowering The Urban Poor to Solve Their Sanitation Problem	Yuyun, I.	2010	Community-based sanitation, wastewater treatment, urban poor, decentralized approach, participatory	Indonesia	National	Evaluation study	2	4

55	Microbiologic effectiveness of boiling and safe water storage in South Sulawesi, Indonesia	Sodha, S. V., Menon, M., Trivedi, K., Ati, A., Figueroa, M. E., Ainslie, R., Wannemuehler, K., and Quick, R	2011	contamination, household, point-of-use, storage, water	South Sulawesi	Provincial	Cross-sectional study	1	3
56	A decision model for selecting sustainable drinking water supply and greywater reuse systems for developing communities with a case study in Cimahi, Indonesia	Henriques, J. J. and Louis, G. E.	2011	Community water management; Developing countries; Drinking water; Greywater; Sanitation; Sustainability	West Java	Provincial	Qualitative study	1	6
57	A sanitary cooperation project in Gorontalo province, Indonesia. Methodology to identifying critical areas, possible strengthening and improving of the health system	Reginato, E., Scali, L., Lizzio, G., Mannocci, A., Rossi, F., and Nocifora, V.	2011	-	Gorontalo	Provincial	Exploratory study	4	5
58	Relationship of the Presence of a Household Improved Latrine with Diarrhea and Under-Five Child Mortality in Indonesia	Semba, R. D., Kraemer, K., Sun, K., de Pee, S., Akhter, N., Moench-Pfanner, R., Rah, J. H., Campbell, A. A., Badham, J., and Bloem, M. W.	2011	-	Banten, Central Java, West Java, East Java, Lampung, South Sulawesi & West Nusa Tenggara	Multi provinces	Cross-sectional study	2	6
59	Confronting culture to overcome sector failure: sanitation in Indonesia	White, W. C.	2011	culture, Indonesia, sanitation, transformational development, values	Indonesia	National	Cross-sectional study	2	4

60	Personal Hygiene and House Sanitation among Children Under Five Years Old with Helminthiasis	Yudhastuti, R., Farid, M., and Lusno, D.	2012	Helminthiasis, Households, Children Under Five Years Old	East Java	Provincial	Case-control study	4	6
61	Factors Associated with Darrhea among Children Under Five Years of Age in Banten Province, Indonesia	Rohmawati, N., Panza, A., and Lertmaharit, S.	2012	factors, diarrhea, children, Indonesia	Banten	Provincial	Case-control study	4	6
62	Use of Household Water Treatment and Safe Storage Methods in Acute Emergency Response: Case Study Results from Nepal, Indonesia, Kenya, and Haiti	Lantagne, D. S., and Clasen, T. F.	2012	-	West Sumatera	Provincial	Cross-sectional study	1	3
63	Relationship between Use of Water from Community-Scale Water Treatment Refill Kiosks and Childhood Diarrhea in Jakarta	Sima, L. C., Desai, M. M., McCarty, K. M., and Elimelech, M.	2012	-	Jakarta	Provincial	Cohort study	1	3
64	The sustainability of urban water supply in low income countries: A livelihoods model	Hadipuro, W., Wiering, M., and Van Naerssen, T.	2013	livelihoods, sustainability, urban, water supply	Central Java	Provincial	A holistic approach	1	6
65	The context and practice of handwashing among new mothers in Serang, Indonesia: A formative research study	Greenland, K., Iradati, E., Ati, A., Maskoen, Y.Y., and Aunger, R.	2013	Hand-washing, Formative research, Behaviour change	Banten	Provincial	Formative research study	3	5
66	Sustainable design of sanitation system based on material and value flow analysis for urban slum in Indonesia	Ushijima, K., Irie, M., Sintawardani, N., Triastuti, J., Hamidah, U., Ishikawa, T., and Funamizu, N.	2013	materialflow, valueflow, resource recyclingsystem, sustainable design, initial cost, urban slum	Jakarta	Provincial	Material flow analysis (MFA) and valueflowanalysis (VFA)	2	3
67	Shaming and sanitation in indonesia: A return to colonial public health practices?	Engel, S., and Susilo, A.	2014	-	Indonesia	National	Qualitative study	2	5

68	Public service provision under conditions of insufficient citizen demand: Insights from the urban sanitation sector in indonesia	Winters, M.S., Karim, A.G., and Martawardaya, B.	2014	Indonesia; Southeast Asia; accountability; local government; public service provision; wastewater sanitation	Indonesia	National	Qualitative study	2	4
69	Economic efficiency of sanitation interventions in Southeast Asia	Hutton, G., Rodriguez, U.-P., Winara, A., Viet-Anh, N., Phyrum, K., Chuan, L., Blackett, I., and Weitz, A.	2014	Asia, benefit-cost ratio, capital cost, economic return, recurrent cost, sanitation	Cambodia, China, Indonesia, Lao PDR, the Philippines and Vietnam	Multi countries	Cost-Benefit analysis	2	1
70	Partial authority allocation of regional water supply system in Indonesia and economic efficiency	Onishi, M., Nababan, M. L., and Kobayashi, K.	2014	-	West Java	Provincial	Evaluation study	1	1
71	Factors involved in sustained use of point-of-use water disinfection methods: a field study from Flores Island, Indonesia	Roma, E., Bond, T., and Jeffrey, P.	2014	chlorine disinfection, Indonesia, solar disinfection technology, sustainability, user acceptance	East Nusa Tenggara	Provincial	Evaluation study	1	6
72	User Perceptions of Shared Sanitation among Rural Households in Indonesia and Bangladesh	Nelson, K. B., Karver, J., Kullman, C., and Graham, J. P.	2014	-	East Java	Provincial	Cross-sectional study	2	5
73	Community-based wastewater treatment systems and water quality of an Indonesian village	Lim, H. S., Lee, L. Y., and Bramono, S. E.	2014	community-based wastewater treatment,	Yogyakarta	Provincial	Case-control study	1	3

				water quality					
74	Sustainability of water supply synchronizing PDAM and HIPPAM in Indonesia	Hermana, J.	2014	downstream, PDAM, upstream, water supply	Indonesia	National	The downstream approach and the upstream approach.	1	4
75	Asymmetric ownership between municipalities in water supply system provision in Indonesia	Nababan, M. L., Onishi, M., and Kobayashi, K.	2014	-	West Java	Provincial	Evaluation study	1	4
76	Aspects of community-based water management and social capital	Syabri, I., Kobayashi, K., Jeong, H., and Ari, I. R. D.	2014	collective action, social capital, community-based water management	Indonesia	National	Exploratory study	1	6
77	Oral and Hand Hygiene Behaviour and Risk Factors among In-School Adolescents in Four Southeast Asian Countries	Peltzer, K., and Pengpid, S.	2014	tooth brushing; hand washing; risk factors; adolescents; Southeast Asian countries	India, Indonesia, Myanmar and Thailand	Multi countries	Cross-sectional study	3	5
78	Network analysis of community based water management	Ari, I. R. D., Jeong, H., Matsushima, K., and Kobayashi, K.	2014	-	East Java	Provincial	Social Network Analysis approach	1	5
79	Planning and installation of a drinking water treatment in Gunungkidul, Java, Indonesia	Fuchs, S., Silva, A., Anggraini, A. K., and Mahdariza, F.	2015	design, slow sand filtration, treatment performance, water treatment	Yogyakarta	Provincial	Cross-sectional study	1	3
80	Morphology, composition and performance of a ceramic filter for household water treatment in Indonesia	Matthies, K., Bitter, H., Deobald, N.,	2015	appropriate technology, bacteria and	West Java	Provincial	Observational study	1	3

		Heinle, M., Diedel, R., Obst, U., and Brenner-Weiss, G.		virus removal, ceramicfilters, leaching of metals, point-of-use,water treatment					
81	Access to Safe Drinking Water and Sanitation in Indonesia	Patunru, A. A.	2015	MDG, Indonesia, water, sanita-tion, diarrhoea	Indonesia	National	Evaluation study	4	6
82	Environmental and Socioeconomics Factors Associated with Cases of Cinical Filariasis in Banyuasin District of South Sumatera, Indonesia	Sapada, I. E., Anwar, C., Salni, and Priadi, D. P.	2015	Filariasis, Environmental, Socioeconomic , Banyuasin district	South Sumatera	Provincial	Case-control study	1	6
83	Understanding modifiable risk factors associated with childhood diarrhea in an eastern Indonesian urban setting	Watson, L., Shibata, T., Ansariadi, Maidin, A., Nikitin, I., and Wilson, J.	2015	childhood diarrhea; mothers' behaviors; safe drinking water: low income	Indonesia	National	Cross-sectional study	1	5
84	Heavy burden of intestinal parasite infections in Kalena Rongo village, a rural area in South West Sumba, eastern part of Indonesia: a Cross-sectional study	Sungkar, S., Pohan, A. P. N., Ramadani, A., Albar, N., Azizah, F., Nugraha, A. R. A., and Wiria, A. E.	2015	Intestinal parasitic infection, Helminthiasis, Hygiene, Intestinal protozoa, Indonesia	West Nusa Tenggara	Provincial	Cross-sectional study	2	2
85	First Indonesian proficiency testing scheme using reference values for cadmium, copper, and iron in drinking water	Ketrin, R., Handayani, E., Mardika, Komalasari, I., and Elishian, C.	2015	Proficiency testing, Reference value, Drinking water, Candidate reference material, z-	Indonesia	National	Randomized Controlled Trial	1	3

				Score					
86	Exploring Determinants of Handwashing with Soap in Indonesia: A Quantitative Analysis	Hirai, M., Graham, J. P., Mattson, K. D., Kelsey, A., Mukherji, S., and Cronin, A. A.	2016	handwashing; hygiene; Indonesia; WASH; open defecation	Indonesia	Provincial	Cross-sectional study	3	5
87	Health risk assessment for exposure to nitrate in drinking water from village wells in Semarang, Indonesia	Sadler, R., Maetam, B., Edokpolo, B., Connell, D., Yu, J., Stewart, D., Park, M.-J., and Gray, D., Laksono, B.	2016	Birth defects; Drinking water; Hazard quotient; Methaemoglobinaemia; Nitrate	Central Java	Provincial	Case-control study	1	2
88	School health promotion: A cross-sectional study on Clean and Healthy Living Program Behavior (CHLB) among Islamic Boarding Schools in Indonesia	Susanto, T., Sulistyorini, L., Wuryaningsih, E. W., and Bahtiar, S.	2016	Clean and Healthy Living Behavior; Health promotion; Islamic boarding school; School health	East Java	Provincial	Cross-sectional study	3	6
89	Prevalence of intestinal protozoan infections and association with hygiene knowledge among primary schoolchildren in salahutu and Leihitu districts, central Maluku regency, Indonesia	Sianturi, M. D. G., Rahakbauw, I. M., Meyanti, F., Kusumasari, R. A., Hartriyanti, Y., Elsa Herdiana, and Murhandarwati, E.	2016	-	Maluku	Provincial	Cross-sectional study	3	2

90	Quantifying accessibility and use of improved sanitation: Towards a comprehensive indicator of the need for sanitation interventions	Park, M. J., Clements, A. C. A., Gray, D. J., Sadler, R., Laksono, B., and Stewart, D. E.	2016	-	Central Java	Provincial	Cross-sectional study	2	5
91	The willingness to pay for in-house piped water in urban and rural Indonesia	Suparman, Y., Folmer, H., and Oud, J. H. L.	2016	Housing hedonic price model, structural equation model (SEM), constrained autoregression, piped water, urban and rural Indonesia	Indonesia	National	Cost-Benefit analysis	1	1
92	Association of safe disposal of child feces and reported diarrhea in Indonesia: Need for stronger focus on a neglected risk	Cronin, A. A., Sebayang, S. K., Torlesse, H., and Nandy, R.	2016	Indonesia; child feces disposal; diarrhea; water; sanitation; hygiene; care practices	Indonesia	National	Cross-sectional study	4	5
93	The coastal community behavior in managing environmental sanitation in Indonesia	Hikmah, Y.	2016		East Nusa Tenggara	Provincial	Case-control study	2	5
94	The influence of iodine supplementation into drinking water as a complement to iodized salt in schoolchildren in a mountainous area of Central Java, Indonesia	Dewi, Y. L. R.	2016	iodinesupplementation, drinking water, schoolchildren, mountainous area, Central Java	Central Java	Provincial	Case-control study	1	5
95	Community development model care for health environment based on water and sanitation (healthy latrine floating eco-	Husaini, Lenie, M., Fauzie, R., Dian, R., Anggun,	2016	E. Coli, River, Water, Health Behavior,	Central Kalimantan		Experimental Method	4	6

	friendly program)	W., Yosua, P., Aan, N., and Tien, Z.		Healthy Latrines Floating					
96	Does piped water improve the welfare of poor households?	Rarassanti, D., Halimatussadiah, A., Hastiadi, F. F., and Muchtar, P. P. S. A.	2016	household welfare, piped water, Propensity Score Matching (PSM).	Banten	Provincial	Cross-sectional study	1	5
97	Drinking water treatment for a rural karst region in Indonesia	Matthies, K., Schott, C., Anggraini, A. K., Silva, A., Diedel, R., Muehlebach, H., Fuchs, S. Obst, U., and Brenner-Weiss, G.	2016	Tropic karst aquifer, Water treatment, Appropriate technology, Slow sand filtration, Ceramic filtration, Point-of-use	Yogyakarta	Provincial	Evaluation study	1	3
98	Determinants of stunting in Indonesian children: evidence from a cross-sectional survey indicate a prominent role for the water, sanitation and hygiene sector in stunting reduction	Torlesse, H., Cronin, A. A., Sebayang, S., Katikana, and Nandy, R.	2016	Indonesia, Stunting, Sanitation, Household water treatment	East Nusa Tenggara	Provincial	Cross-sectional study	4	5
99	Exploring Determinants of Handwashing with Soap in Indonesia: A Quantitative Analysis	Hirai, M., Graham, J. P., Mattson, K. D., Kelsey, A., Mukherji, S., and Cronin, A. A.	2016	handwashing; hygiene; Indonesia; WASH; open defecation	East Nusa Tenggara, South Sulawesi & Papua	Multi provinces	Cross-sectional study	3	5
100	Molecular survey of Blastocystis sp. from humans and associated animals in an Indonesian community with poor hygiene	Yoshikawa, H., Tokoro, M., Nagamoto, T., Arayama, S., Asih, P. B. S. Rozi, I. E., and	2016	Blastocystis sp.; Host specificity; Molecular epidemiology; Subtype;	East Nusa Tenggara	Provincial	Cross-sectional study	3	5

		Syafruddin, D.		Zoonotic transmission.					
101	Transfer of adapted water supply technologies through a demonstration and teaching facility	Nestmann, F., Oberle, P., Ikhwan, M., Stoffel, D., Blass, H. J., Toews, D., and Schmidt, S.	2016	Water supply , Adapted technology , Capacity development , Pump as turbine , Wood stave penstock	Yogyakarta	Provincial	Evaluation study	1	3
102	A new approach to nationwide sanitation planning for developing countries: Case study of Indonesia	Kerstens, S. M., Spiller, M., Leusbrock, I., and Zeeman, G.	2016	GIS; Investment and operational costs; Nationwide planning; Solid waste; Wastewater.	Indonesia	National	Cross-sectional study	1	6
103	Enabling factors for sustaining open defecation-free communities in rural Indonesia: A cross-sectional study	Odagiri, M., Muhammad, Z., Cronin, A.A., Gnilo, M.E., Mardikanto, A.K., Umam, K., and Asamou, Y.T.	2017	Indonesia; Open Defecation Free (ODF) sustainability; community approaches to total sanitation (CATS); latrine use; social norms.	Nusa Tenggara Timur	Provincial	Cross-sectional study	2	5
104	The switch to refillable bottled water in Indonesia: A serious health risk	Komarulzaman, A., De Jong, E., and Smits, J.	2017	drinking water, Indonesia, LMIC, piped water, refillable bottled water	Indonesia	National	Qualitative study	1	6

105	The effect of physical accessibility and service level of water supply on economic accessibility: a case study of Bandung City, Indonesia	Nastiti, A., Sudradjat, A., Geerling, G.W., Smits, A.J.M., Roosmini, D., and Muntalif, B.S.	2017	Affrodability, equitable access, mitigation, physical and economic accessibility, SDGs, Indonesia	West Java	Provincial	Empirical Study	1	1
106	Piloting water quality testing coupled with a national socioeconomic survey in Yogyakarta province, Indonesia, towards tracking of Sustainable Development Goal 6	Cronin, A.A., Odagiri, M., Arsyad, B., Nuryetty, M.T., Amannullah, G., Santoso, H., Darundiyah, K., and Nasution, N.	2017	Drinking water safety; Hygiene; Indonesia; Sanitation; Sustainable development goals	Yogyakarta	Provincial	Case-control study	1	6
107	Determinants of stunting among children in urban families in palu, Indonesia	Rahman, N., Napirah, M.R., Nadila, D., and Bohari	2017	Stunted children, urban area, family income, environmental sanitation, exclusive breast feeding, maternal age	Central Sulawesi	Provincial	Case-control study	2	5
108	Clean water, sanitation and diarrhoea in Indonesia: Effects of household and community factors	Komarulzaman, A., Smits, J., and de Jong, E.	2017	Diarrhoea; Indonesia; drinking water; multilevel analysis; sanitation	Indonesia	National	Case-control study	4	5
109	Sanitation value chains in low density settings in Indonesia and Vietnam: Impetus for a rethink to achieve pro-poor outcomes	Willetts, J., Gero, A., Susamto, A.A., Sanjaya, R., Trieu, T.D., Murta, J., and Carrard, N.	2017	market-based approaches, pro-poor, rural sanitation, supply chain, value chain	East Nusa Tenggara	Provincial	Quantitative and qualitative methods	2	5

110	Improving water, sanitation, and hygiene in schools in Indonesia: A cross-sectional assessment on sustaining infrastructural and behavioral interventions	Karon, A.J., Cronin, A.A., Cronk, R., and Hendrawan, R.	2017	Hygiene; Indonesia; Sanitation; Sustainable Development Goals (SDGs); WASH in schools; Water	Indonesia	National	Cross-sectional study	4	5
111	'Fit for school' - a school-based water, sanitation and hygiene programme to improve child health: Results from a longitudinal study in Cambodia, Indonesia and Lao PDR	Duijster, D., Monse, B., Dimaisip-Nabuab, J., Djuharnoko, P., Heinrich-Weltzien, R., Hobdell, M., Kromeyer-Hauschild, K., Kunthearith, Y., Mijares-Majini, M.C., Siegmund, N., Soukhanouvong, P., and Benzian, H.	2017	Dental caries; Deworming; Handwashing; School health; Soil-transmitted helminth infection; Toothbrushing; Underweight; Water sanitation and hygiene.	West Java	Provincial	Longitudional study	4	5
112	Coping with poor water supply in peri-urban Bandung, Indonesia: towards a framework for understanding risks and aversion behaviours	Nastiti, A., Muntalif, B.S., Roosmini, D., Sudradjat, A., Meijerink, S.V., and Smits, A.J.M.	2017	affordability, aversion behaviours, Bandung, continuity, dimensions of access, quality, quantity, risk, water supply	West Java	Provincial	Case-control study	1	5
113	The relationship between hygiene sanitation and contamination of Escherichia coli in snacks in elementary schools in Depok, Indonesia	Dinasari, H., and Wispriyono, B.	2017	E.Coli, Elementary School, Hygiene, Sanitation, Snacks	West Java	Provincial	Cross-sectional study	4	5

114	How important is culture to sanitation uptake? The influence of local values in rural Bali	Dwipayanti, N., Rutherford, S., Phung, D., and Chu, C.	2017	Comprehensive Assessment; Local Values; Rural Bali; Sanitation Uptake; Sustainability	Bali	Provincial	Randomized controlled trial	2	5
115	Household water supply strategies in urban Bandung, Indonesia: Findings and implications for future water access reporting	Muntalif, B.S., Nastiti, A., Roosmini, D., Sudradjat, A., Meijerink, S.V., and Smits, A.J.M.	2017	household water treatment and storage; MDGs; mixed water sources; monitoring; SDGs; water supply.	West Java	Provincial	Cross-sectional study	1	6
116	Citizen engagement to sustaining community-based rural water supply in Indonesia	Kasri, R.Y., Wirutomo, P., Kusnoputranto, H., and Moersidik, S.S.	2017	Service delivery, Citizen engagement, Sustainable development, Rural water supply	West Java & West Sumatera	Multi provinces	Evaluation study	1	4
117	Livelihood structures and household access to drinking water sources in West Ungaran Sub-District Semarang Regency Central Java Province Indonesia	Dan Sunarko, P.H.	2017	Livelihood Structure, Agriculture, Non-Agriculture, Household Access, Drinking Water Sources	Central Java	Provincial	Quantitative and descriptive methods	1	2
118	Traveler's diarrhea risk factors on foreign tourists in Denpasar Bali-Indonesia	Ani, L. S. and Suwiyoga, K.	2017	traveler’s diarrhea, case control, risk factors, tourists	Bali	Provincial	Case-control study	3	6

119	Women's Participation in a Rural Water Supply and Sanitation Project: A Case Study in Jorong Kampung Baru, Nagari Gantung Ciri, Kubung Subdistrict, Solok, West Sumatra, Indonesia	Yuerlita	2017	Women's participation, Gender equity, Policy, Institutional arrangement, Sociocultural aspect, Religious beliefs and practices	West Sumatera	Provincial	Exploratory study with an appropriate research design	4	4
120	Intervening with healthcare workers' hand hygiene compliance, knowledge, and perception in a limited-resource hospital in Indonesia: a randomized controlled trial study	Santosaningsih, D., Erikawati, D., Santoso, S., Noorhamdani, N., Ratridewi, I., Candradikusuma, D., Chozin, I. N., Huwae, T. E. C. J., van der Donk, G., van Boven, E., Holt, A. F. V. I. V., H. A., and Severin, J. A.	2017	Hand hygiene, Healthcare-associated infections, Indonesia	East Java	Provincial	Randomized controlled trial	3	5
121	Urban-Rural Interrelations in Water Resource Management: Problems and Factors Affecting the Sustainability of the Drinking Water Supply in the City of Bandung, Indonesia	Parikesit, Husodo, T., Okubo, S., Herwanto, T., Badri, I., Gunawan, R., Megantara, E. N., Muhammad, D., and Takeuchi, K.	2017	Catchment Bandung Drinking water Intensive agriculture Land conversion	West Java	Provincial	multidisciplinary study	1	2
122	Soil Transmitted Helminth Infections in Medan: a cross-sectional study of the correlation between the infection and nutritional status among elementary school children	Darlan, D. M., Alexandra, T. S., and Tala, Z. Z.	2017	nutritional status, school-aged children, STH infection, underweight	North Sumatera	Provincial	Cross-sectional study	2	5

123	Nitrate in drinking water and risk of colorectal cancer in Yogyakarta, Indonesia	Fathmawati, Fachiroh, J., Gravitiani, E., Sarto, and Husodo, A. H.	2017	-	Yogyakarta	Provincial	Case-control study	1	5
124	Comparison of boiling and chlorination on the quality of stored drinking water and childhood diarrhoea in Indonesian households	Fagerli, K., Trivedi, K. K., Sodha, S. V., Blanton, E., Ati, A., Nguyen, T., Delea, K. C., Ainslie, R., Figueroa, M. E., Kim, S., and Quick, R.	2017	Escherichia coli (E. coli), gastrointestinal disease, water (quality), water-borne infections	Banten	Provincial	Case-control study	1	3
125	`Bad' piped water and other perceptual drivers of bottled water consumption in Indonesia	Prasetiawan, T., Nastiti, A., and Muntalif, B. S.	2017	-	Indonesia	National	Qualitative study	1	5
126	The role of bottled drinking water in achieving SDG 6.1: an analysis of affordability and equity from Jakarta, Indonesia	Walter, C. T., Kooy, M., and Prabaharyaka, I.	2017	affordability, bottled water, drinking water, equity, Jakarta, SDG 6	Jakarta	Provincial	Cross-sectional study	1	6
127	Evaluation of acceptance of a composting toilet prototype for people in slum area in Indonesia	Ito, R., Tanie, M., Ushijima, K., Nilawati, D., Sintawardani, N., and Funamizu, N.	2017	Muslim; Feces–urine–water separation; Solid–liquid separator; Acceptance; Interface design	Indonesia	National	Experimental Method	2	3
128	Critical Systems Thinking Review on Decentralized Drinking Water Management in Nauli City, Indonesia	Simbolon, J.	2017	decentralisatio n; sustainability; systems	North Sumatera	Provincial	Ideas, Editorials, Opinions	1	4

				thinking; water					
129	The Association Between Sanitation, Hygiene, and Stunting in Children Under Two-Years (An Analysis of Indonesia's Basic Health Research, 2013)	Badriyah, L. and Syafiq, A.	2017	children under two-years; hygiene; sanitation; stunting	Indonesia	National	Cross-sectional study	4	5
130	Equity implications for sanitation from recent health and nutrition evidence	Cronin, A. A., Gnilo, M. E., Odagiri, M., and Wijesekera, S.	2017	Sanitation, Health impact, Equity, SDG target 6, Wash, Indonesia	Indonesia	National	Qualitative study	2	5
131	Sanitation entrepreneurship in rural Indonesia: a closer look	Murta, J.C.D., Willetts, J.R.M., and Triwahyudi, W.	2018	Sanitation entrepreneurship, Sanitation business, Social enterprise, Privatesector, Enterprise development	Indonesia	National	Political economy analysis frame-work	2	1
132	Water, sanitation, and hygiene services in public health-care facilities in Indonesia: Adoption of world health organization/united nations children's fund service ladders to national data sets for a sustainable development goal baseline assessment	Odagiri, M., Cahyorini, Azhar, K., Cronin, A.A., Gressando, Y., Hidayat, I., Utami, W., Widowati, K., Roshita, A., Soeharno, R., Warouw, S.P., and Ardhiantie	2018	-	Indonesia	National	Evaluation study	4	4
133	Stunting trends and associated factors among Indonesian children aged 0-23 months: Evidence from Indonesian Family Life Surveys (IFLS) 2000, 2007 and 2014	Hanifah, L., Wulansari, R., Meiandayati, R., and Achadi, E.L.	2018	Stunting, stunting trend in Indonesia, undernutrition, IFLS	Indonesia	National	Trend Analysis	2	5

134	Cost minimization of raw water source by integrated water supply systems (a case study for Bandung, Indonesia)	Afiatun, E., Notodarmojo, S., Effendi, A.J., and Sidarto, K.A.	2018	Cost minimization; Integrated water supply systems; Optimization; Superstructure model	West Java	Provincial	Case-control study	1	3
135	Risk analysis of drinking water process in drinking water treatment using fuzzy FMEA Approach	Rimantho, D. and Hatta, M.	2018	Clean water; FMEA fuzzy; Processing; Quality; Risk identification and evaluation	West Java	Provincial	Fishbone method approach and risk analyze using the FMEA fuzzy model	1	4
136	High prevalence of cognitive impairment among students near mount Merapi: A case study	Nguyen, H. T., Wijayanti, L. W., Kim, K., Lee, W. K., Lee, C. H., and Shin, K	2018	Impaired cognition; Intellectual dis-abilities; Malnutrition; Stunting	Yogyakarta	Provincial	Cross-sectional study	4	5
137	Liquidity Constraints and Willingness to Pay for Solar Lamps and Water Filters in Jakarta	Lensink, R., Raster, T., and Timmer, A.	2018	economics; microfinance; willingness to pay; water filters; solar lamps; BDM auction; Indonesia	Jakarta	Provincial	Quantitative study	1	1
138	Community-based water supplies in Cikarang, Indonesia: are they sustainable?	Roekmi, R. A. K., Baskaran, K., and Chua, L. H. C.	2018	Community-based water supply; common pool resources; developing country; Cikarang; Indonesia	West Java	Provincial	Evaluation study	1	4

139	Analysing piped water service provider performance based on consumer perceptions	Roekmi, R. A. K., Chua, L. H. C., Baskaran, and Kanagaratnam	2018	Performance indicator, Consumer perception, Consumer perception	West Java	Provincial	Assessment methode	1	4
140	Domestic water supply, residential water use behaviour, and household willingness to pay: The case of Banda Aceh, Indonesia after ten years since the 2004 Indian Ocean Tsunami	Jiang, Y. and Rohendi, A.	2018	Banda Aceh; Use behaviour; Utility; Water supply; Willingness to pay	Aceh	Provincial	Cross-sectional study	1	1
141	Water Sources Quality in Urban Slum Settlement along the Contaminated River Basin in Indonesia: Application of Quantitative Microbial Risk Assessment	Sari, S. Y. I., Sunjaya, D. K., Shimizu-Furusawa, H., Watanabe, Chiho., and Raksanagara, A. S.	2018	-	West Java	Provincial	Cross-sectional study	1	5
142	Reducing Methicillin-Resistant Staphylococcus Aureus (MRSA) cross-infection through hand hygiene improvement in Indonesian intensive tertiary care hospital	Dahesihdewi, A., Dwiprahasto, I., Wimbarti, S., and Mulyono, B.	2018	compliance, hand hygiene, intensive care, MRSA cross transmission	Yogyakarta	Provincial	Quasi-experimental & qualitative study	3	3
143	Child Malnutrition in Indonesia: Can Education, Sanitation and Healthcare Augment the Role of Income?	De Silva, I. and Sumarto, S	2018	Child nutrition, Malnutrition, Stunting, Quantile regression, Indonesia.	Indonesia	National	Quantile regression	4	4
144	Shadow puppets and neglected diseases: Evaluating a health promotion performance in rural Indonesia	Kurscheid, J., Bendrups, D., Susilo, J., Williams, C., Amaral, S., Laksono, B., Stewart, D. E.,	2018	Health promotion; Indonesia; Knowledge and behaviours; Shadow	Central Java	Provincial	Qualitative study	4	5

		and Gray, D. J.		puppetry; Soil-transmitted helminths					
145	Subnational regional inequality in access to improved drinking water and sanitation in Indonesia: results from the 2015 Indonesian National Socioeconomic Survey (SUSENAS)	Afifah, T., Nuryetty, M. T., Cahyorini, Musadad, D. A., Schlotheuber, A., Bergen, N., and Johnston, R.	2018	Indonesia, water, sanitation, health inequality, health equity	Indonesia	National	Randomized Controlled Trial	4	5
146	Determinants of toilet ownership among rural households in six eastern districts of Indonesia	Hirai, M., Kelsey, A., Mattson, K., Cronin, A. A., Mukerji, S., and Graham, J. P.	2018	Indonesia; Open defecation; Sanitation; Social norms	Papua, South Sulawesi, East Nusa Tenggara,	Multi provinces	Cross-sectional study	2	5
147	Correlation between food hygiene sanitation and escherichia coli (E.coli) contamination on snacks sold around elementary school in Jatiasih subdistrict, Bekasi Indonesia	Hazairin, C. A., Made Djaja, I., and Hartono, B.	2018	E.coli contamination; Hygiene sanitation; Snacks at elementary schools	West Java	Provincial	Cross-sectional study	4	5
148	The influence of nurse's knowledge level on behaviour changes, attitude and 5 moments of hand hygiene compliance	Soesanto, D.	2018	Attitudes; Behaviour; Compliance; Level of knowledge; Nursing home	East Java	Provincial	Cross-sectional study	3	5
149	Physical environment of home affecting the infection of helminthiasis among toddlers in rural areas	Gunawan, A. T., Triyantoro, B., Subagyo, A., Mulidah, S., and Marsum Kusumawati, S Rajiani, I	2018	Helminthiasis; Home; Infection; Physical environment; Toddler	Central Java	Provincial	Cross-sectional study	2	6

150	Health risk assessment of coliform bacteria contamination in the dug well water with qmra to predict public health risk in small island, makassar	Birawida, A. B., Selomo, M., Mallongi, A., and Ismita, U. W. Suriah	2018	-	South Sulawesi	Provincial	Case-control study	1	2
151	The level of environmental sanitation and the incidence of tuberculosis in Jember and Situbondo, Indonesia	Ma'rufi, I., Khorir, A., Ali, K., and Nugroho, H. S. W.	2018	Tuberculosis, Hygiene, Snitation, Environment	East Java	Provincial	Cross-sectional study	2	6
152	Implementation of electre methods in determining for recipient candidate for pamsimas program in district pringsewu	Irviani, R., Mukodimah, S., Muslihudin, M., and Trisnawati.	2018	Community-Based Water Drinking Supply and Sanitati; ELECTRE; Pringsewu; Ranking	Central Java	Provincial	Evaluation study	4	4
153	Health risk assessment and vulnerability of children in flood prone area of Makassar	Syamsuar, Haris, A., and Suwahyuni, R.	2018	Coliform; Dug well water; QMRA	South Sulawesi	Provincial	Case-control study	1	3
154	Effect of education health wash hands of changes in knowledge and attitude of women taking care of children of Diarrhea in hospital Wangaya Denpasar	Sipahutar, I. E., Sulisnadewi, N. L. K., Wedri, N. M., Candra, I. W., Lestari, A. S., Yasa, I. D. P. G. P., and Runiari, N. N.	2018	Attitude; Diarrhea; Health education; Knowledge	Bali	Provincial	Case-control study	3	5
155	Analysis on quality of food sanitation hygiene and escherichia coli (E. coli) contamination at restaurants around commuter line stations in central Jakarta, 2018	Ratnasari, D. A., Djaja, I. M., and Hartono, B.	2018	E.Coli bacteria quality, Hygiene sanitation, Restaurants	Jakarta	Provincial	Cross-sectional study	4	5
156	Changing rural communities behavior towards safe water and improved sanitation in indonesia	Kasri, R. Y., Kusnoputranto, H., Wirutomo, P., and Moersidik, S.	2018	Behavior change; Environmental health; Evidence-	West Sumatera	Provincial	Case-control study	4	5

				based; Sanitarian; Sustainability					
157	The analysis of fecal coliforms and coliform total in wells water at the tourism area of Sanur	Jirna, I. N. and Mallongi, A.	2018	fecal coliform, total coliform, well water	Bali	Provincial	Cross-sectional study	2	2
158	Development of clean water distribution network capacity by using watercad	Bisri, M., Sisinggih, D., and Putra, W. D.	2018	Clean water; Pipe network; Water-CAD v.8 XM Edition	East Java	Provincial	Quantitative study	1	3
159	Study on Communal Wastewater Treatment Plants (CWWTPs) in Gresik, Indonesia	Soedjono, E. S., Fitriani, N. Setiawan, A., Mulia, G. J. T., and Ningsih, D. A.	2019	Wastewater treatment plants	East Java	Provincial	Quantitative study	2	3
160	Effects of water and health on primary school enrolment and absenteeism in Indonesia	Komarulzaman, A., De Jong, E., and Smits, J	2019	Diarrhoea; Drinking water; Indonesia; Panel; School absenteeism; School attendance	Indonesia	National	Longitudinal Study	1	5
161	Participation and Power Dynamics Between International Non-Governmental Organisations and Local Partners: A Rural Water Case Study in Indonesia	Cunningham, I., Willetts, J., Winterford, K., and Foster, T.	2019	Rural water supply, power, participation, community-based management, Indonesia	Bali	Provincial	A diffracted power frame	1	4
162	The Reliability Study of Raw Water Sources in The Development of Potable Water Supply Systems in Indonesia	Afifah, E., Sabar, A., Wulandari, S., and Marselina, M.	2019	Dependable discharge, Hydrology, Markov model, Rainfall, Raw	Banten, West Kalimantan, and Bangka Belitung	Multi provinces	Cross-sectional study	1	3

				water					
163	Risk factors for undernutrition and diarrhea prevalence in an urban slum in Indonesia: Focus on water, sanitation, and hygiene	Otsuka, Y., Agestika, L., Widyarani, Sintawardani, N., and Yamauchi, T.	2019	-	West Java	Provincial	Cross-sectional study	4	5
164	The determinant factor of nurse's hand hygiene adherence in Indonesia	Handiyani, H., Ikegawa, M., Hariyati, Rr. T. S., Ito, M., and Amirulloh, F.	2019	Hand hygiene adherence; Nurse education; Nurse's attitude; The head of ward's support	Jakarta	Provincial	Cross-sectional study	3	5
165	Effects of the implementation of a progressive tariffs policy on water management in DKI Jakarta, Indonesia	Suratin, A., Triakuntini, E., and Herdiansyah, H.	2019	progressive water tariff, urban water supply, water use efficiency, water demand management	Jakarta	Provincial	Quantitative approach	1	1
166	Water access in post-tsunami Indonesia	Burrows, M.	2019	Disaster; Recovery; Water; Infrastructure; Resources; Vulnerabilit; Inequality; Resilience; Introduction	Aceh	Provincial	Randomized control trial	1	3

167	Towards A Situated Urban Political Ecology Analysis of Packaged Drinking Water Supply	Kooy, M. and Walter, C. T.	2019	urban water infrastructure; political ecology; water governance; water quality; packaged drinking water (PDW); bottled water; Jakarta; Indonesia	Jakarta	Provincial	Qualitative study	1	6
168	A situational analysis of a healthy school canteen development program: Lessons learned from a selected group of primary schools in Jakarta, Indonesia	Meiyetriani, E., Februhartanty, J., Iswarawanti, D. N., and Sudibya, A. R. P.	2019	Food hygiene; Jakarta; School canteen; School nutrition	Jakarta	Provincial	Cross-sectional study	2	2
169	Domestic Water Adequacy of Surakarta, Indonesia: Is it Prone to Vulnerability?	Rahayu, P., Rini, E. F., and Soedwiwahjono	2019	Urbanization, medium-sized city, domestic water supply, vulnerability, Surakarta	Central Java	Provincial	Cross-sectional study	1	6
170	Not built to last: Improving legal and institutional arrangements for community-based water and sanitation service delivery in Indonesia	Al'Afghani, M. M., Kohlitz, J., and Willetts, J.	2019	Community, institutions, water supply, sanitation, sustainability, Indonesia	Indonesia	National	Empirical study	4	4
171	Adherence to preoperative hand hygiene and sterile gowning technique among consultant surgeons, surgical residents, and nurses: A pilot study at an academic medical center in Indonesia	Handaya, A. Y. and Werdana, V. A. P.	2019	owning technique, Hand hygiene, Donning the gloves, Abdominal surgery	Yogyakarta	Provincial	Cross-sectional study	3	3

172	Water quality trend assessment in Jakarta: A rapidly growing Asian megacity	Luo, P., Kang, S., Apip, Zhou, M., Lyu, J., Aisyah, S., Binaya, M., Regmi, R. K., and Nover, D.	2019	-	Jakarta	Provincial	Cohort study	1	3
173	A multifaceted hand hygiene improvement program on the intensive care units of the National Referral Hospital of Indonesia in Jakarta	Saharman, Y. R., Aoulad Fares, D., El-Atmani, S., Sedono, R., Aditianingsih, D., Karuniawati, A., Van Rosmalen, J., Verbrugh, H. A., Severin, J. A.,	2019	Hand hygiene, Quality improvement, Guideline adherence, Intensive care unit, Indonesia	Jakarta	Provincial	observational, prospective, before-and-after intervention study	3	5
174	Sanitizing Jakarta: decolonizing planning and kampung imaginary	Putri, P. W.	2019	Urban sanitation, water management, urban planning, colonial modernity, community development, Jakarta, urban kampungs	Jakarta	Provincial	Ideas, Editorials, Opinions	2	5
175	Photovoice interactive media improves the personal hygiene of teenage students at pesantren school in Tangerang	Samsuni, S., Mulyono, S., Wiarsih, W., and Heni Kusumawardani, L.	2019	Personal hygiene; Photovoice interactive media; Teenager	Banten	Provincial	Quasi-experiment	3	5
176	Water quality mapping of piped water supply in Malang City - Indonesia	Rofida, R., Fitriani, N., Indarko, D. G., Yuniarto, A.,	2019	District meter areas	East Java	Provincial	Case-control study	1	3

		Marsono, B. D., and Soedjono, E. S.							
177	Comprehensive assessment of handwashing and faecal contamination among elementary school children in an urban slum of Indonesia	Otsuka, Y., Agestika, L., Harada, H., Sriwuryandari, L., Sintawardani, N., and Yamauchi, T.	2019	awareness; bidonville; child; connaissance; contamination fécale; enfant; faecal contamination; handwashing; knowledge; lavage des mains; sensibilisation; urban slum.	West Java	Provincial	Cross-sectional study	4	5
178	Scaling up sanitation: Evidence from an RCT in Indonesia	Cameron, L., Olivia, S., and Shah, M.	2019	Health; Impact evaluation; Sanitation; Scale up; Social capital	Indonesia	National	Randomized control trial	2	6
179	Prevalence and risk factors of soil-transmitted helminthiasis among school children living in an agricultural area of North Sumatera, Indonesia	Pasaribu, A. P., Alam, A., Sembiring, K., Pasaribu, S., and Setiabudi, D.	2019	Soil-transmitted helminth, Risk factor, School children, Indonesia	North Sumatera	Provincial	Cross-sectional study	4	5
180	City Sanitation Planning Through a Political Economy Lens	Abeysuriya, K., Willetts, J., Carrard, N., and Kome, A.	2019	Urban sanitation, sanitation planning, political economy, developing countries, Southeast Asia	Indonesia, the Philippines, Malaysia	Multi countries	Evaluation study	2	1

181	Impact of the "balatrine" intervention on soil-transmitted helminth infections in central Java, Indonesia: A pilot study	Gray, D. J., Kurscheid, J. M., Park, M. J., Laksono, B., Wang, D., Clements, A. C. A., Hadisaputro, S., Sadler, R., and Stewart, D. E.	2019	water, sanitation and hygiene (WASH), latrine intervention, soil-transmitted helminths, Indonesia	Central Java	Provincial	Faecal flotation diagnostic method	4	3
182	The struggle for water in indonesia: The role of women and children as household water fetcher	Irianti, S. and Prasetyoputra, P	2019	Gender; Indonesia; Water collection; Water fetcher; Water source	Indonesia	National	Cross-sectional study	1	6
183	Cultural determinants of sanitation uptake and sustainability: local values and traditional roles in rural Bali, Indonesia	Dwipayanti, N. M. U., Rutherford, S., and and Chu, C.	2019	cultural determinants, local values, rural Bali, sanitation uptake, sustainability,traditional roles	Bali	Provincial	Qualitative and descriptive approaches based on the grounded theory	2	5
184	Determinants of adolescent shortness in Tanjungsari, West Java, Indonesia	Sasongko, E. P. S., Ariyanto, E. F., Indraswari, N., Rachmi, C. N., and Alisjahbana, A.	2019	birth weight, environment, hygiene, maternal literacy, shortness, 12-year-old children, water	West Java	Provincial	Longitudinal Study	2	6
185	Factors affecting bacteria contamination among food handlers at a public hospital in South Sumatra, Indonesia	Utari, S., Hermansyah, Saleh, I., and Pambayun, R.	2019	Eschericia Coli, rectal swab, hygiene, infection, bivariate analysis	South Sumatera	Provincial	Bivariate analysis	3	5

186	Detection of Escherichia coli O157:H7 and Shiga toxin 2a gene in pork, pig feces, and clean water at Jagalan slaughterhouse in Surakarta, Central Java Province, Indonesia	Goma, M. K. E., Indraswari, A., Haryanto, A., and Widiasih, D. A.	2019	Escherichia coli O157:H7; Feces; Pork; Shiga toxin 2a; Slaughterhouse ; Water	Central Java	Provincial	Cohort study	1	2
187	Evaluation of Sanitation Risk Index to Achieve Sustainable Development Goals 2020 in Sanitation Sector	Arimbawa, I. G., Santosa, F. R. E., Nilowardono, Mudjanarko, S., and Wiwoho	2019	Millenium Development Goals (MDGs), Sustainable Development Goals (SDGs), Sanitation Sector	East Java	Provincial	Environmenta l Health Risk Assessment (EHRA) approach	2	5
188	Water and sanitation program in decentralised eastern Indonesia: The roles of community and social dynamics	Susilo, A., Vidyattama, Y., and Eva Wishanti, D. A. P.	2019	Decentralisatio n; Developing countries; Indonesia; Water and sanitation	Indonesia	National	Ideas, Editorials, Opinions	4	4
189	An integrated system approach to characterise a drinking water infrastructure system	Kloosterman, R. A., and van der Hoek, J. P.,	2019	system of systems; SoS; decision-making; complexity; drinking water; critical infrastructure; water resources; social-ecological system; technical infrastructure; social-technical system; long-	Central Java	Provincial	the SoPhyTech infra framework	1	3

				term decisions making; long life time assets; Semarang; Vitens.					
190	Institutional inertia: challenges in urban water management on the path towards a water-sensitive Surabaya, Indonesia	Kosters, M., Bichai, F., and Schwartz, K.	2019	Water governance; water-sensitive city; urban water management; institutional challenges; modern infrastructural ideal; Surabaya	East Java	Provincial	Evaluation study	1	4
191	Water consumption assessment in Asian chemical industries supply chains based on input-output analysis and one-way analysis of variance	Shafiei, M., Moosavirad, S., Hamed, Azimifard, A., and Biglari, S.	2019	Water consumption management . Supply chain management . Chemical industry . Input-output analysis . ANOVA	Indonesia	National	input-output analysis	1	5
192	Hygiene and sanitation challenge for covid-19 prevention in Indonesia	Purnama, S. G., and Susanna, D.	2019	COVID-19; Hygiene; Indonesia; Sanitation	Indonesia	National	Qualitative study	4	6

193	Automation of drinking water treatment systems in rural area	Nursubiyantoro, E., Ismianti, and Wibowo, A. W. A.	2019	Automation, Design, Prototype, Rural area, Water treatment system	Yogyakarta	Provincial	Action research design	1	3
194	Analysis of free residual chlorine in drinking water distribution systems in ore processing industry	Susanto, A., Amrina, U., Purwanto, P., Putro, E. K., Yochu, W. E., and Wilmot, J. C.	2019	Drinking Water Distribution Systems; EPAnet; Free Residual Chlorine; Water quality modeling	Papua	Provincial	Observational study	1	3
195	The influence of personal hygiene and healthy lifestyle on environmental sanitation	Dewi, Y. S., and Kurniawan, D.	2019	personal hygiene, healthy lifestyle, environmental sanitation	West Java	Provincial	Cross-sectional study	4	5
196	Determination of mercury (Hg) risk level (rq) with exposure through fish and drinking water consumption in Bulawa sub-district, Bone bolango district, Gorontalo province, Indonesia	Rahmadhani, T. N., Tualeka, A., R., Rahmawat, P., Russen, S. S., Wahy, A., Ahsa, and Singga, S.	2019	Mercury (Hg); Mine; Risk Level (RQ); Society	Gorontalo	Provincial	Observational study	1	6
197	Hygiene and sanitation management of drinking water refill depots for feasibility consumption in Kendari City, Indonesia	Nurlila, R. U., La Fua, J., Yuli Munandar, K., Muh Sainal, A., Sahlan, L., and Mallongi, A.	2019	Hygiene, Sanitation, Management of Drinking Water, and Refill Depots	Southeast Sulawesi	Provincial	Descriptive study	4	5

198	Analysis of water sources availability and water quality in dry and rainy season in dry land areas, North Gresik, Indonesia	Kusdarini, E., Suyadi, S., Yanuwiadi, B., and Hakim, L.	2019	Availability; Dry land; Lake; Quality; Well water	East Java	Provincial	Cross-sectional study	1	3
199	The effect of the community led total sanitation (Clts) method on the event of diarrhea in balita in banjar district	Husaini, Marlinae, L., Akba Agustriyono, R., Aprilia, R., Maimunah, Ulfah, N., and Zubaidah, T.	2019	Flies density, Environmental factors, Sanitation facilities, Sanitation behavior.	South Kalimantan	Provincial	Cross-sectional study	2	5
200	Child health care practices and stunting in children aged 12-36 months in jember regency of Indonesia	Kusumawardani, D. A., Irawan, R., and Purnomo, W.	2019	Child health care practices; Drinking watter; Imunization; Sanitation; Stunting	East Java	Provincial	Case-control study	4	5
201	Effect of educational factors on the use of clean water in rural communities in the dry tropical areas, East Nusa Tenggara – Indonesia	Messakh, J. J., and Zakarias, H. C.	2019	Dry tropic climate; East Nusa Tenggara; Educational factors; Rural water; Semi-arid	East Nusa Tenggara	Provincial	Empirical Research	1	5
202	Community led total sanitation (CLTS) in Cikupa village and Teluknaga village in Tangerang, Indonesia	Kusumaningtiar, D. A., and Vionalita, G.	2019	Clts; Diarrhea; Environmental ; Indonesia; Sanitation	West Java	Provincial	Cross-sectional study	3	2
203	Latrine use and associated factors among rural community in Indonesia	Yulyani, V., Dina Dwi, N., and Kurnia, D.	2019	Latrine Use, Open Defecation, Tanggamus Regency	Lampung	Provincial	Cross-sectional study	2	5

204	Microbiological quality, hygiene, and sanitation of the production processes of a traditional beverage at tourism areas in Bali	Sugianti, G. R., Wirawan, I. M. A., and Utami, N. W. A.	2019	Environmental health; Escherichia coli; Food safety; Foodborne diseases; Healthy tourism.	Bali	Provincial	Cross-sectional study	4	3
205	Failure reduction on the operational treatment of drinking water refills in Wonokromo Subdistrict, Surabaya, Indonesia	Mappangara, H., and Karnaningroem, N.	2019	Refill drinking water station; Structural Equation Modeling; fishbone diagram; Likert scale	East Java	Provincial	Structural Equation Modeling (SEM) method	1	6
206	Sanitation, open defecation, and Diarrhea in Tangerang, Banten, Indonesia, in early 2017: A cross-sectional epidemiological study	Tutuanita, N. N. Y., and Zakianis	2019		Banten	Provincial	Cross-sectional study	2	4
207	The relationship of gender, school sanitation and personal hygiene with helminthiasis at juhar karo regency in North Sumatera Province, Indonesia	Agustaria, G. Fazidah, A. S., and Nurmaini, N.	2019	Helminthiasis, Gender, School sanitation, Personal hygiene	North Sumatera	Provincial	Cross-sectional study	3	5
208	Enhancing health quality of islamic boarding school students through hygiene practices in Depok and Banten, Indonesia	Rianti, E., Triwinarto, A., Rodoni, A., and Elina	2019	Boarding school health post; Personal hygiene; Santri health	West Java	Provincial	Cross-sectional study	3	6
209	The relationship between environmental sanitation to the incidence of hepatitis a in rural areas of Central Java, Indonesia	Widyanto, T., Marsum, Choerul, A. M., Subinarto, Fikri, A., Asep Tata, G., and Rajiani, I.	2019	Hepatitis A, environmental sanitation, Health Community Center	Central Java	Provincial	Case-control study	2	2

210	Compliance in maintaining hand cleaning on health care workers in neonatology unit in tertiary referral hospital indonesia: The usage of cctv for supervision	Taryana, A. M., Sampurna, M. T. A., and Sari, G. M.	2019	Cctv; Hand hygiene; Health care workers; Neonatology units; Referral hospital	East Java	Provincial	Cross-sectional study	3	5
211	phenomenology of unsustainable sanitation in developing countries: Grounded theory methods in coastal area of Indonesia	Suning and Pungut	2019	Coastal communities, Communal latrines, Defecation habits, Policy, Sanitation	East Java	Provincial	Qualitative Studies	2	5
212	Photovoice interactive media improves the personal hygiene of teenage students at pesantren school in Tangerang	Samsuni, S., Mulyono, S., Wiarsih, W., and Heni, K. L.	2019	Personal hygiene; Photovoice interactive media; Teenager	West Java	Provincial	Quasi-experimental study	3	3
213	Rainwater as a Source of Drinking Water: Health Impacts and Rainwater Treatment	Khayan, K., Heru Husodo, A., Astuti, I., Sudarmadji, S., and Sugandawaty, D. T.	2019	-	West Kalimantan	Provincial	Cross-sectional study	1	3
214	Relationship between sanitation hygiene and health care with healthy family security of the family of smokers at berastagi subdistrict	Nurmaini, N., and Sudaryati, E.	2019	Health care; Healthy family security; Hygiene sanitation; Smoker	North Sumatera	Provincial	Case-control study	4	5
215	Domestic Water Adequacy of Surakarta, Indonesia: Is it Prone to Vulnerability?	Rahayu, P., Rini, E. F., and Soedwiwahjono	2019	Urbanization, medium-sized city, domestic water supply, vulnerability, Surakarta	Central Java	Provincial	Statistical descriptive analysis	1	4

216	Safe concentration of lead in community drinking water in the Tapak river area, Tugu city district, Semarang	Safitri, M. R., Tualeka, A. R., Raharjo, P., Rahmawati, P., Russeng, S. S., Wahyu, A., and Ahsan	2019	Lead (Pb); Safe concentration; Tapak river water	Central Java	Provincial	an observational study with non reactive research using reference dose (RfD) calculation and LOAEL	1	2
217	Impact of complementary foods and environmental sanitation on the incidence of diarrhea in children aged 6-24 months in sidoarjo, Indonesia	Krisnana, I., Pradanie, R., and Mustika, D. A.	2020	Complementary food,environmentalsanitation, diarrhea,childrenaged6-24months.	East Java	Provincial	Cross-sectional study	2	2
218	A hierarchical Bayesian Belief Network model of household water treatment behaviour in a suburban area: A case study of Palu- Indonesia	Daniel, D., Sirait, M., and Pande, S.	2020	-	Central Sulawesi	Provincial	Cross-sectional study	1	5
219	Analysis of factors related to personal behaviour of hygiene on the ship crew in pt salam pasific indonesia lines surabaya	Raoef, F. P., Has, E. M. M., and Yasmara, D.	2020	Knowledge, Attitude, Friend support, Facilities and infrastructure, Personal hygiene, Crew members	East Java	Provincial	Cross-sectional study	3	5
220	Evaluation of a package of behaviour change interventions (baduta program) to improve maternal and child nutrition in east Java, Indonesia: Protocol for an impact study	Dibley, M. J., Alam, A., Fahmida, U., Ariawan, I., Titaley, C. R., Htet, M. K., Damayanti, R., Li, M., Sutrisna, A., and Ferguson, E.	2020	Diet; Feeding behavior; Food; Growth disorders; Infant; Nutrition; Nutrition during pregnancy; Undernutrition	East Java	Provincial	Randomized Controlled Trial	3	4

				; Water treatment					
221	Identifying the green supply chain for environmental sanitation management in Banda Aceh, Indonesia	Yusuf, R., Maimun, Sanusi, Saputra, J., and Gani, S. A.	2020	Green Supply Chain Management, Environmental Sanitation,Waste	Aceh	Provincial	Quantitative study	2	6
222	Improved sanitation is associated with reduced child stunting amongst Indonesian children under 3 years of age	Rah, J. H., Sukotjo, S., Badgaiyan, N., Cronin, A. A., and Torlesse, H.	2020	Anaemia; Indonesia; WASH; sanitation; stunting	Papua, Central Java, and West Nusa Tenggara	Multi provinces	Randomized Controlled Trial	2	6
223	Predictors of healthy lifestyle in the covid-19 pandemic period in east Java, Indonesia	Laksono, A. D., Ibad, M., Herwant, Y. T., Sarweni, K. P., Geno, R. A. P., Nugraheni, E., and Wulandari, R. D.	2020	health education, healthy lifestyle, health behavior, health promotion, COVID-19.	East Java	Provincial	Cross-sectional study	3	5
224	Achieving the Sustainable Development Goals for water and sanitation in Indonesia – Results from a five-year (2013–2017) large-scale effectiveness evaluation	Odagiri, M., Cronin, A. A., Thomas, A., Kurniawan, Afrianto, M., Zainal, M., Setiabudi, W., Gnilo, M., Emerson, Badloe, C., Virgiyanti, T. D., Nurali, I. A., Wahanudin, L.,	2020	-	East Nusa Tenggara, South Sulawesi, and Papua	Multi provinces	Cohort Study	4	4

		Mardikanto, A., and Pronyk, P.,							
225	Solution to water scarcity in the eastern indonesia: A case study of the lembata regency	Masduqi, A., Nugroho, A. R., and Wilujeng, S. A.	2020	Lembata Regency, Reservoir, Supply-demand analysis, Water scarcity	East Nusa Tenggara	Provincial	Supply-demand analysis	1	2
226	A national communication campaign in Indonesia is associated with improved WASH-related knowledge and behaviors in Indonesian mothers	Hanson, C., Allen, E., Fullmer, M., O'brien, R., Dearden, K., Garn, J., Rachmi, C. N., Glenn, J., West, J., Crookston, B., and Hall, P.	2020	Behavior; Interpersonal communication strategies; Knowledge; Media messages; WASH	Indonesia	National	Cross-sectional study	4	5
227	Utilisation of the family latrine post declaration ODF	Ahyanti, M., Rosita, Y., and Yushananta, P.	2020	Diarrhea, ODF, Desa Pemanggilan	Lampung	Provincial	Qualitative Studies	2	5
228	Automation of drinking water treatment systems in rural area	Nursubiyantoro, E., Ismianti, and Wibowo, A. W. A.	2020	Automation; Design; Prototype; Rural area; Water treatment system	Yogyakarta	Provincial	Literature Study	1	3

229	Home sanitation and personal hygiene relation to leprosy	Hayana, Marlina, H., Gumayesty, Y., and Sulastri	2020	House Sanitation, Personal Hygiene, Occurrence.	Riau	Provincial	Cross-sectional study	4	5
230	Soil-transmitted helminth infections and taeniasis on Samosir Island, Indonesia	Wandra, T., Darlan, D. M., Yulfi, H., Purba, I. E., Sato, M. O., Budke, C. M., and Ito, A.	2020	Indonesia; Risk factors; Samosir Island; Soil-transmitted helminth infections; Taeniasis	North Sumatera	Provincial	Longitudional study	4	5
231	Risk behavior of tiom community related with helminthiasis at lanny jaya district, Papua province, indonesia	Putro, G., Ristrini., Sukoco, N. E. W., and Dewi, E. R.	2020	Barapen Ceremony; Clean and healthy lifestyle; Helminthiasis; Papua Indonesia; Tiom	Papua	Provincial	descriptive retrospective research	4	5
232	Water and sanitation program in decentralised eastern Indonesia: The roles of community and social dynamics	Susilo, A., Vidyattama, Y., and Eva Wishanti, D. A. P.	2020	Water and sanitation; decentralisation; developing countries; Indonesia.	East Nusa Tenggara	Provincial	Evaluation study	4	5
233	Training intervention to improve hygiene practices in Islamic boarding school in Yogyakarta, Indonesia: A mixed-method study	Widyasari, V., Prabandari, Y. S., and Utarini, A.	2020	-	Yogyakarta	Provincial	A mixed-methods exploratory sequential design	3	5
234	Risk factors of STH infections in children aged 6-12 years in sub-villages II and IV Manusak village of east Kupang district - Kupang Regency year 2019	Djuma, A. W. and Olin, W. Pan, I M	2020	Characteristics, Behavior, Sanitation	East Nusa Tenggara	Provincial	Cross-sectional study	2	5

235	Analysis of risk factors in the post-disaster of diarrhea in Donggala district, Indonesia	Wahyuni, R. D., Mutiarasari, D., Miranti, Demak, I., Puspasari K., Pasinringi, S. A., and Mallongi, A.	2020	Diarrhea; Natural disasters; Risk factors; Sanitation	Central Sulawesi	Provincial	Cross-sectional study	2	2
236	Contributing factors of stunted growth among toddlers in makassar city: A qualitative study	Tahangnacca, M., Ridwan, A., Ansariadi, and Syam, A.	2020	Feeding practices; Infectious diseases; Socio-economic status; Stunted growth; Toddlers	South Sulawesi	Provincial	Qualitative study	1	6
237	Behavior of schmutzdecke with varied filtration rates of slow sand filter to remove total coliforms	Matuzahroh, N., Fitriani, N., Ardiyanti, P. E., Kuncoro, E. P., Budiyanto, W. D., Isnadina, D. R. M., Wahyudianto, F. E., and Radin Mohamed, R. M. S.	2020	Chemical engineering; Drinking water; Environmental analysis; Environmental engineering; Environmental science; Schmutzdecke; Slow sand filter; Total coliforms; Waste; Water quality; Water treatment	East Java	Provincial	Cross-Over Studies	1	3
238	Short communication: Prevalence and risk factors of soil-transmitted helminth infection among farmers in gelgel village, Klungkung District, Bali, Indonesia	Apsari, P. I. B., Indraningrat, A. A. G., Arwati, H., and Dachlan, Y. P.	2020	Bali, farmers, prevalence, risk factors, soil-transmitted helminth	Bali	Provincial	Cross-sectional study	2	5

239	Determinants of diarrhea among children under two years old in Indonesia	Santika, N. K. A., Efendi, F., Rachmawati, P. D., Has, E. M. M., Kusnanto, K., and Astutik, E.	2020	Child; Demographic and Health Survey; Diarrhea	Indonesia	National	Cross-sectional study	3	6
240	Association between toilet availability and handwashing habits and the incidence of stunting in young children in Tanjung Pinang City, Indonesia	Ahmadi, Sulistyorini, L., Azizah, R., and Oktarizal, H.	2020	Handwashing habits, Stunting, Toilet availability	Riau Archipelago	Provincial	Cross-sectional study	3	5
241	Personal Impact of Media Publicity During Covid-19 Pandemic and Awareness Among Generation Z in Jakarta, Indonesia	Roselina, E. and Asmiyanto, T.	2020	COVID-19; Generation Z; Large-scale Social Restrictions (PSBB); Media Publicity; Personal impact	Jakarta	Provincial	Case-control Study	3	5
242	Sustainable water services fee using a system dynamics approach in Jatigede reservoir	Muldianto, H., Andawayanti, U., Suhartanto, E., and Soetopo, W.	2020	Water Service Fee, System dynamics, sustainable management, Multipurpose reservoir.	West Java	Provincial	system dynamics approach	1	1
243	Risk factors for stunting among children in Banggai Regency, Indonesia	Gani, A. A., Widasari, L., Otoluwa, A. S., Hadju, V., Palutturi, S., Thaha, A. R., and Manti B, S.	2020	Age; Children; Exclusive breastfeeding; Latrine; Stunting; Water source	Central Sulawesi	Provincial	Cross-sectional study	2	5
244	Hygiene and sanitation challenge for covid-19 prevention in Indonesia	Purnama, S. G., and Susanna, D.	2020	COVID-19, hygiene, sanitation, Indonesia	Indonesia	National	Qualitative Studies	4	5

245	Epidemiology of soil-transmitted helminth infections in semarang, central java, indonesia	Kurscheid, J., Laksono, B., Park, M. J., Clements, A. C. A., Sadler, R., McCarthy, J. S., Nery, S. V., Soares-Magalhaes, R., Halton, K., Hadisaputro, S., Richardson, A., Indjein, L., Wangdi, K., Stewart, D E., and Gray, D. J.	2020	-	Central Java	Provincial	Cross-sectional study	2	5
246	Home environmental health: Relationship with stunting in bandar lampung	Masra, F. and Helmy, H.	2020	Environmental Health of House, Stunting.	Lampung	Provincial	Quantitative methods	1	5
247	Effect of drinking water habits and oral hygiene status in elementary school children: A Quasi experimental study	Setyowati, D., Cahyani, D., Heroesoebekti, R., and Ramadhani, A.	2020	Children Oral Hygiene; Debris Index; Drinking Behavior; Mineral Water; Oral Hygiene	East Java	Provincial	Quasi-experimental study	4	5
248	Microbial risk assessment (MRA) as a method of assessment for drinking water refll in pattinggaloang district of Makassar city	Baharuddin, A. and Ichsan, M.	2020	Drink water; E.coli; MRA; Microbial risk	North Sulawesi	Provincial	Descriptive with Microbial Risk Assessment (MRA)	1	5
249	Soil-Transmitted Helminth Infections, Anemia, and Undernutrition Among School-Children in An Elementary School in North Jakarta, Indonesia	Sari, M. P., Nathasaria, T., Majawati, E. S., and Pangaribuan, H. U.	2020	Children, nutritional status, North Jakarta, soil-transmitted	Jakarta	Provincial	Cross-sectional study	4	5

				helminths					
250	A Bayesian Belief Network model to link sanitary inspection data to drinking water quality in a medium resource setting in rural Indonesia	Daniel, D., Iswarani, W. P., Pande, S., and Rietveld, L.	2020	-	East Nusa Tenggara	Provincial	Cross-sectional study	4	5
251	Evaluation of Program for Overcoming Intestinal Worm Infections among Children	Febriyanti, H. and Idris, H.	2020	drug distribution, evaluation, worm infection	South Sumatera	Provincial	Qualitative research design	2	4
252	The effect of socio-economic characteristics on the use of household water treatment via psychosocial factors: a mediation analysis	Daniel, D., Pande, S., and Rietveld, L.	2020	household water treatment, mediation analysis, psychosocial factors, human behaviour	East Nusa Tenggara	Provincial	Cross-sectional study	1	5
253	Investigating Community Preferences in Fulfilling Domestic Water Needs to Improve Public Water Service Provision A Case Study in Kota Metro, Lampung Province, Indonesia	Sugiyono and Dewancker, B. J.	2020	Community preference, correspondence analysis, domestic water use, Kota Metro	Lampung	Provincial	Case-control Study	1	6
254	Correlation between Personal Hygiene, Household Hygiene, and Atopic Dermatitis in Elementary School Children in Indonesia	Bahari, M. I. Y., and Paramita, D. A.,	2020	Atopic dermatitis, household hygiene, personal hygiene	North Sumatera	Provincial	Cross-sectional study	3	5
255	Public Toilets, Stink and Power	Martosenjoyo, T., Naping, H., Rahim, M.R.., and Lampe, M.	2020	Public Toilets; Architecture; Stink; Power; Status	South Sulawesi	Provincial	Qualitative Studies	2	5

256	Sustaining Community-Scale Sanitation Services: Co-management by Local Government and Low-Income Communities in Indonesia	Willetts, J., Mills, F., and Al'Afghani, M.	2020	co-management, sanitation, sustainability, local government, institutional arrangements	Indonesia	National	Qualitative Studies	2	4
257	Assessing the Impact and Equity of an Integrated Rural Sanitation Approach: A Longitudinal Evaluation in 11 Sub-Saharan Africa and Asian Countries	Apanga, P. A., Garn, J. V., Sakas, Z., and Freeman, M. C.	2020	sanitation; coverage; equity; WASH; vulnerable	Indonesia	National	Cross-sectional study	2	4
258	Solar energy-based water treatment system applicable to the remote areas: Case of Indonesia	Wibowo, A. I. and Chang, K. C.	2020	developing country, Indonesia, remote area, solar energy, solar thermal, water treatment system	South Sulawesi	Provincial	Quantitative research method	1	6
259	Health risk assessment for exposure to nitrate in drinking water in Central Java, Indonesia	Lowe, C., Kurscheid, J., Lal, A., Sadler, R., Kelly, M., Stewart, D., Laksono, B., Amaral, S., and Gray, D.	2021	Birth defects; Drinking water; Health risk assessment; Indonesia; Nitrate	Central Java	Provincial	Cross-sectional study	1	3
260	Modelling behavioural change from sea-based `helicopter latrines' to land-based shared improved latrines in the Demaan, Jepara, Indonesia	Sunarti, S., Helmi, M., Widjajanti, R., Purwanto, A., and Amellia	2021	behaviour setting, community empowerment, sanitation, slums, sustainable behaviour	Central Java	Provincial	Qualitative Studies	2	6

261	Birth Weight and Length Associated with Stunting among Children Under-Five in Indonesia	Lukman, T. N. E., Anwar, F., Riyadi, H., Harjomidjojo, H., and Martianto, D.,	2021	birth weight, birth length, social-economic, stunting	South Sulawesi	Provincial	Cross-sectional study	4	5
262	A system dynamics model of the community-based rural drinking water supply program (Pamsimas) in Indonesia	Daniel, D., Prawira, J., Djono, T. P. A., Subandriyo, S., Rezagama, A., and Purwanto, A.	2021	rural drinking water supply; system dynamics; Indonesia; PAMSIMAS; sustainability	Central Java	Provincial	Causal Loop Diagram	1	6
263	Challenges for national deworming policy in Indonesia: experience from Bandung district West Java province	Adrizain, R., Setiabudi, D., Faridah, L., Fauziah, N., and Setiabudiawan, B.	2021	Bandung; Deworming; Indonesia; Policy; Stunting	West Java	Provincial	Qualitative Studies	2	4
264	E-Monitoring the Vulnerability of Malnutrition of Children using The Topsis Method	Nurhayati, S. and Lubis, R.	2021	Child's nutritional status, Fuzzy topsis, Malnutrition factors.	Indonesia	National	Topsis Method	4	6
265	Interaction of factors influencing the sustainability of water, sanitation, and hygiene (Wash) services in rural indonesia: Evidence from small surveys of wash-related stakeholders in indonesia	Daniel, D., Djohan, D., and Nastiti, A.	2021	Causal loop diagram; Indonesia; Sanitation; Sustainability; Water	Indonesia	National	Causal Loop Diagram	4	4
266	Understanding Period Poverty: Socio-Economic Inequalities in Menstrual Hygiene Management in Eight Low- and Middle-Income Countries	Rossouw, L. and Ross, H.	2021	menstrual health; menstrual hygiene management; inequality; water and sanitation; gender;	Indonesia	National	Empirical Study	4	5

				environmental health; sanitary pads					
267	Risk factors of stunting in indonesian children aged 1 to 60 months	Wicaksono, R. A., Arto, K. S., Mutiara, E., Deliana, M., Lubis, M., and Batubara, J R. L.	2021	Growth diagrams of Indonesian children; Risk factors; Stunting	Aceh	Provincial	Case-control study	4	6
268	Use of technology to access health information/services and subsequent association with wash (water access, sanitation, and hygiene) knowledge and behaviors among women with children under 2 years of age in Indonesia: Cross-sectional study	Niedfeldt, H. J., Beckstead, E., Chahalis, E., Jensen, M., Reher, B., Torres, S., Rachmi, C. N., Jusril, H., Hall, C., West, J. H., and Crookston, B. T.	2021	Defecation; Handwashing; Stunting; Technology; WASH	Indonesia	National	Cross-sectional study	4	5
269	Hookworm infection still prevalent in the less developed urban area in jakarta, Indonesia	Surja, S. S., Ali, S., Ajisuksmo, C., Pramono, H., Iustitiani, N. S D., Celine, and Cindy.	2021	Child; Environment; Helminthiasis; Poverty	Jakarta	Provincial	Observational Study	2	3
270	Knowledge, attitude and practice of hygiene and sanitation among food-handlers in a psychiatric hospital in Indonesia - A mixed method study	Palupi, I. R., Fitasari, R. P., and Utami, F. A.	2021	Attitude and practice; Food-handler; Hygiene and sanitation; Knowledge; Psychiatric hospital	Indonesia	National	A mixed method study	4	5

271	Use of technology to access health information/services and subsequent association with wash (water access, sanitation, and hygiene) knowledge and behaviors among women with children under 2 years of age in Indonesia: Cross-sectional study	Niedfeldt, H. J., Beckstead, E., Chahalis, E., Jensen, M., Reher, B., Torres, S., Rachmi, C. N., Jusril, H., Hall, C., West, J. H., and Crookston, B. T.	2021	Defecation; Handwashing; Stunting; Technology; WASH	Indonesia	National	Cross-sectional study	4	5
272	Evaluation of feeding practices for infants and children (PMBA) for stunting children in Lombok	Nurbaiti, L., Taslim, N., Hatta, M., and Bukhari, A.	2021	Evaluation; Feeding practices; PMBA; Stunting; Toodlers	West Nusa Tenggara	Provincial	Cross-sectional study	3	5

PRISMA 2020 Checklist

Section and Topic	Item #	Checklist item	Location where item is reported
**TITLE**			
Title	1	Identify the report as a systematic review.	V
**ABSTRACT**			
Abstract	2	See the PRISMA 2020 for Abstracts checklist.	V
**INTRODUCTION**			
Rationale	3	Describe the rationale for the review in the context of existing knowledge.	V
Objectives	4	Provide an explicit statement of the objective(s) or question(s) the review addresses.	V
**METHODS**			
Eligibility criteria	5	Specify the inclusion and exclusion criteria for the review and how studies were grouped for the syntheses.	V
Information sources	6	Specify all databases, registers, websites, organisations, reference lists and other sources searched or consulted to identify studies. Specify the date when each source was last searched or consulted.	V
Search strategy	7	Present the full search strategies for all databases, registers and websites, including any filters and limits used.	V
Selection process	8	Specify the methods used to decide whether a study met the inclusion criteria of the review, including how many reviewers screened each record and each report retrieved, whether they worked independently, and if applicable, details of automation tools used in the process.	V
Data collection process	9	Specify the methods used to collect data from reports, including how many reviewers collected data from each report, whether they worked independently, any processes for obtaining or confirming data from study investigators, and if applicable, details of automation tools used in the process.	V
Data items	10a	List and define all outcomes for which data were sought. Specify whether all results that were compatible with each outcome domain in each study were sought (e.g. for all measures, time points, analyses), and if not, the methods used to decide which results to collect.	V
	10b	List and define all other variables for which data were sought (e.g. participant and intervention characteristics, funding sources). Describe any assumptions made about any missing or unclear information.	V
Study risk of bias assessment	11	Specify the methods used to assess risk of bias in the included studies, including details of the tool(s) used, how many reviewers assessed each study and whether they worked independently, and if applicable, details of automation tools used in the process.	V
Effect measures	12	Specify for each outcome the effect measure(s) (e.g. risk ratio, mean difference) used in the synthesis or presentation of results.	
Synthesis methods	13a	Describe the processes used to decide which studies were eligible for each synthesis (e.g. tabulating the study intervention characteristics and comparing against the planned groups for each synthesis (item #5)).	V
	13b	Describe any methods required to prepare the data for presentation or synthesis, such as handling of missing summary statistics, or data conversions.	
	13c	Describe any methods used to tabulate or visually display results of individual studies and syntheses.	V
	13d	Describe any methods used to synthesize results and provide a rationale for the choice(s). If meta-analysis was performed, describe the model(s), method(s) to identify the presence and extent of statistical heterogeneity, and software package(s) used.	
	13e	Describe any methods used to explore possible causes of heterogeneity among study results (e.g. subgroup analysis, meta-regression).	
	13f	Describe any sensitivity analyses conducted to assess robustness of the synthesized results.	
Reporting bias assessment	14	Describe any methods used to assess risk of bias due to missing results in a synthesis (arising from reporting biases).	
Certainty	15	Describe any methods used to assess certainty (or confidence) in the body of evidence for an outcome.	

PRISMA 2020 Checklist

Section and Topic	Item #	Checklist item	Location where item is reported
assessment			
**RESULTS**			
Study selection	16a	Describe the results of the search and selection process, from the number of records identified in the search to the number of studies included in the review, ideally using a flow diagram.	V
	16b	Cite studies that might appear to meet the inclusion criteria, but which were excluded, and explain why they were excluded.	V
Study characteristics	17	Cite each included study and present its characteristics.	V
Risk of bias in studies	18	Present assessments of risk of bias for each included study.	
Results of individual studies	19	For all outcomes, present, for each study: (a) summary statistics for each group (where appropriate) and (b) an effect estimate and its precision (e.g. confidence/credible interval), ideally using structured tables or plots.	
Results of syntheses	20a	For each synthesis, briefly summarise the characteristics and risk of bias among contributing studies.	
	20b	Present results of all statistical syntheses conducted. If meta-analysis was done, present for each the summary estimate and its precision (e.g. confidence/credible interval) and measures of statistical heterogeneity. If comparing groups, describe the direction of the effect.	
	20c	Present results of all investigations of possible causes of heterogeneity among study results.	V
	20d	Present results of all sensitivity analyses conducted to assess the robustness of the synthesized results.	
Reporting biases	21	Present assessments of risk of bias due to missing results (arising from reporting biases) for each synthesis assessed.	
Certainty of evidence	22	Present assessments of certainty (or confidence) in the body of evidence for each outcome assessed.	
**DISCUSSION**			
Discussion	23a	Provide a general interpretation of the results in the context of other evidence.	V
	23b	Discuss any limitations of the evidence included in the review.	V
	23c	Discuss any limitations of the review processes used.	V
	23d	Discuss implications of the results for practice, policy, and future research.	V
**OTHER INFORMATION**			
Registration and protocol	24a	Provide registration information for the review, including register name and registration number, or state that the review was not registered.	
	24b	Indicate where the review protocol can be accessed, or state that a protocol was not prepared.	V
	24c	Describe and explain any amendments to information provided at registration or in the protocol.	
Support	25	Describe sources of financial or non-financial support for the review, and the role of the funders or sponsors in the review.	V
Competing interests	26	Declare any competing interests of review authors.	V
Availability of data, code and other materials	27	Report which of the following are publicly available and where they can be found: template data collection forms; data extracted from included studies; data used for all analyses; analytic code; any other materials used in the review.	V

From: Page MJ, McKenzie JE, Bossuyt PM, Boutron I, Hoffmann TC, Mulrow CD, et al. The PRISMA 2020 statement: an updated guideline for reporting systematic reviews. BMJ 2021;372:n71. doi:

PRISMA 2020 Checklist

10.1136/bmj.n71

For more information, visit: http://www.prisma-statement.org/
